# Dissection of brassinosteroid-regulated proteins in rice embryos during germination by quantitative proteomics

**DOI:** 10.1038/srep34583

**Published:** 2016-10-05

**Authors:** Qian-Feng Li, Min Xiong, Peng Xu, Li-Chun Huang, Chang-Quan Zhang, Qiao-Quan Liu

**Affiliations:** 1 Key Laboratory of Crop Genetics and Physiology of Jiangsu Province/Key Laboratory of Plant Functional Genomics of the Ministry of Education/Co-Innovation Center for Modern Production Technology of Grain Crops, College of Agriculture, Yangzhou University, Yangzhou 225009, People’s Republic of China

## Abstract

Brassinosteroids (BRs), essential plant-specific steroidal hormones, function in a wide spectrum of plant growth and development events, including seed germination. Rice is not only a monocotyledonous model plant but also one of the most important staple food crops of human beings. Rice seed germination is a decisive event for the next-generation of plant growth and successful seed germination is critical for rice yield. However, little is known about the molecular mechanisms on how BR modulates seed germination in rice. In the present study, we used isobaric tags for relative and absolute quantification (iTRAQ) based proteomic approach to study BR-regulated proteome during the early stage of seed germination. The results showed that more than 800 BR-responsive proteins were identified, including 88 reliable target proteins responsive to stimuli of both BR-deficiency and BR-insensitivity. Moreover, 90% of the 88 target proteins shared a similar expression change pattern. Gene ontology and string analysis indicated that ribosomal structural proteins, as well as proteins involved in protein biosynthesis and carbohydrate metabolisms were highly clustered. These findings not only enrich BR-regulated protein database in rice seeds, but also allow us to gain novel insights into the molecular mechanism of BR regulated seed germination.

Brassinosteroids (BRs), a group of plant-specific polyhydroxylated steroidal hormones, are involved in a wide spectrum of plant growth and developmental processes, including cell elongation, seed germination, photomorphogenesis, etc^1^,^2^,^3^. BR deficient or insensitive mutants have been identified in a number of plant species, such as Arabidopsis, rice, maize, pea, tomato, barley^4^. The mutants display various growth defects, including dwarfism, dark green leaves, delayed flowering, male sterility, and photomorphogenesis in the dark^5^. A combination of molecular genetic and proteomic studies in Arabidopsis contributes to the dissection of BR signaling pathway, which is considered as one of the best-understood signal transduction pathways in plants at present^6^.

In brief, BR signals are perceived by the trans-membrane leucine-rich-repeat containing receptor-like kinase (LRR-RLK) BRI1^7^. BR binding triggers the fast activation of BRI1’s intracellular kinase domain via phosphorylation and homodimerization^8^. Then a downstream phosphorylation and dephosphorylation dependent signal transduction cascade is activated by BRI1, leading to inactivation of BIN2, a central negative regulator of BR signaling pathway^9^. Therefore, BZR1 and BES1, two key transcription factors, are dephosphorylated and activated^10^,^11^. Dephosphorylated BZR1 and BES1 translocate into the nuclear to activate the transcription of downstream target genes by binding the BRRE elements and E-box of their promoters^12^,^13^,^14^,^15^.

Due to the striking advances in deconstructing BR signaling pathway in Arabidopsis, great progress has also been made in elucidating how BR regulates diverse developmental and physiological events, especially the prominent role of BR in inspiriting cell elongation^16^. The promotion effect of BR on seed germination is long been observed. Seed germination is one of the most complex and critical physiological processes in plant growth and development. Previous reports showed that BR can rescue the germination phenotype of severe GA-deficient and GA-insensitive mutants in Arabidopsis^2^. In addition, germination of BR-deficient and BR-insensitive mutants is more strongly inhibited by ABA compared to their wild-type counterparts, and therefore BR can partially overcome the inhibition effect on germination by ABA^17^,^18^. Recently, Hu and Yu^19^ demonstrated that BIN2, the key negative regulator in the BR signaling pathway, could directly interact with ABI5, a bZIP transcription factor in ABA pathway, to antagonize the inhibitory roles of ABA on seed germination. Application of BR on mutants *gpa1*, *gcr1* and *mft* further confirmed the notion that BR played an important role in promoting seed germination^20^,^21^,^22^. However, these progresses were mainly achieved in Arabidopsis and only linear connections between BR and several seed germination related genes have been set up. However, no systemic networks have been established to illustrate how BR regulates rice seed germination to date.

Generally, rice seed germination could be divided into three different phases: Phase I is the first stage of seed germination characterized by a quick water-uptake and the onset of mRNA biosynthesis^23^. Phase II is the most important stage for seed germination, during which stage a number of germination events occurred, including metabolism reactivation, reserve mobilization, coleoptile elongation, cell wall loosening and repair of cell structure, etc. Phase III is another rapid water-uptake stage accompanied by initiation of cell division and seedling establishment, radical protrusion, as well as recovering of TCA and aerobic respiration^24^. Different from Arabidopsis seed, rice seed has a relative small embryo and a dominant endosperm for nutrient-storage. The embryo, containing most of the genetic information of rice, plays a decisive role during seed germination. Thus, quantitative proteomic study of rice embryo is a powerful and efficient way to uncover BR-regulated seed germination network.

In the present study, we used the seeds of mutant *d61-1*^25^ and brassinazole (BRZ) treated Nipponbare, representing BR insensitive and BR deficient germination conditions, respectively. After seeds germinated in darkness for 36 hours, we separated the embryos and extracted total proteins for an iTRAQ-based quantitative proteomic analysis, thus to unravel BR-regulated target proteins during rice seed germination. The results would give some insights into our understanding of the regulatory roles of BR in rice seed germination.

## Results

### Germination process of rice seeds with BR-exhausted

**Figure 1:**
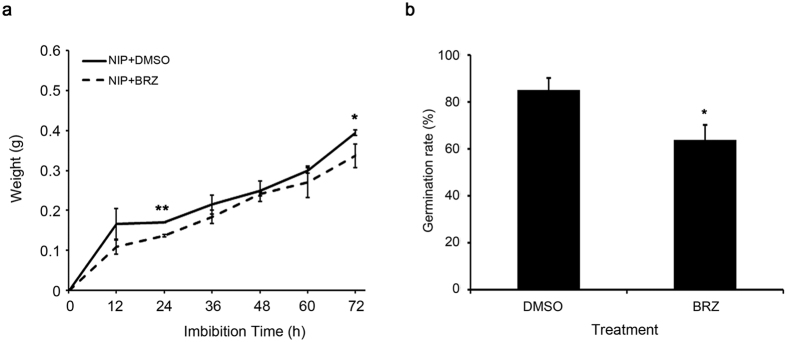
Effects of BRZ on rice seed germination. (**a**) Changes in seed weight for 30 rice seeds during germination. (**b**) Rice seed germination rates after 72 h of imbibition with BRZ and DMSO treatment, respectively. Each value is the mean of three biological replicates. The asterisks indicate a significantly difference between BRZ and DMSO treated seeds by the Student’s t test (*p < 0.05, **p < 0.01).

In general, fresh weight increment of germinated seeds follows a classic triphasic model, including the rapid water uptake phase (phase I), the plateau phase (phase II) and the post-germinative growth phase (phase III)^26^. Moreover, increment in seed fresh weight mainly come from endosperm at phase I and embryo at phase III, respectively^27^. Our preliminary germination result showed that the embryo size of Nipponbare increased constantly within the first 48 h of imbibition and the protruded radicle and coleoptile could be clearly seen and recorded around 72 h (Supplementary Fig. 1). To further explore the role of BR in seed germination process, BR biosynthesis inhibitor BRZ was applied to mimic BR-deficient germination conditions. Nipponbare seeds were imbibed in Milli-Q water supplemented with BRZ or DMSO in darkness and the increment of seed fresh weight was monitored every 12 h, from 0 h to 72 h. Generally, BRZ-treated and mock-treated seeds shared a similar increment pattern of seed fresh weight. However, extent of increment was smaller in BRZ-treated seeds than that in the mock-treated controls (Fig. 1a). Moreover, germination rate of BRZ treated seeds was lower than that of mock treated controls (Fig. 1b), implying that BR may involve in the water-uptake, germination rate or some other biological activities during rice seed germination.

### Changes of protein abundance in rice embryos during early stage of seed germination

Proteome study is a powerful tool to identify BR-responsive proteins during seed germination. Thus, non-gel-based iTRAQ technique was used to quantify the expression change of target proteins *in vivo*. Embryos from three different rice materials were used in the present study. First, Nipponbare was treated with BRZ or its mock DMSO, respectively. BRZ treatment was used to mimic the BR-deficient germination condition. Second, a mild BR-insensitive mutant *d61-1* was used, representing seeds with BR signaling pathway partially disrupted. *d61-1* is defective in *OsBRI1* gene, exhibiting dwarfism phenotype, male sterility, delayed flowering, and pleotropic defects in cell elongation, similar to the Arabidopsis *bri1* mutant^25^. After seeds germinated in darkness for 36 hours, belonging to phase II stage of seed germination, embryos were separated from the seeds and total proteins were extracted for following iTRAQ proteomic analysis (Supplementary Fig. 2). Total proteomes from different samples were labeled by iTRAQ, the isobaric chemical labeling reagents, and quantified for identifying proteins with abundance changed. Our primary objective was to identify BR-regulated proteins in the embryos during the early stage of seed germination. Specifically, protein abundances between Nipponbare/BRZ and Nipponbare/DMSO as well as between *d61-1*/DMSO and Nipponbare/DMSO were carefully compared, respectively. Therefore, after protein identification by MS/MS, a large number of differentially expressed proteins were identified according to the result of protein ratio distribution analysis (Supplementary Fig. 3a,b). Generally, number of proteins with abundance changed was much larger in *d61-1* mutant than that in the BRZ-treated wild-type controls. Specifically, total 232 and 608 proteins were identified in response to BRZ treatment or *OsBRI1* mutation, respectively, based on the 1.2-fold-change criterion with P < 0.05. Among the proteins responsive to BRZ treatment, 125 proteins were up-regulated and 107 were down-regulated (Supplementary Table S1 and S2). As to the 608 proteins responsive to *OsBRI1* mutation, 325 proteins were up-regulated and 283 proteins were down-regulated (Supplementary Table S3 and S4). The ratio for all the detected proteins with abundance changed varied from 0.19 to 4.34, and 777 out of the 840 identified proteins had abundance ratios within the range of 0.5 to 2. Therefore, it could be concluded that small changes in protein abundance were observed in our iTRAQ data.

### Functional analysis of 88 reliable BR-responsive proteins

**Figure 2:**
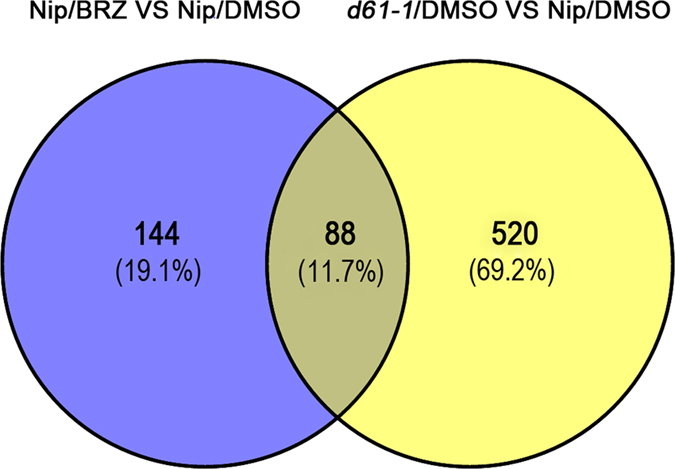
Venn diagram illustrating the number of identified proteins with abundance changed in the embryos of both BRZ treated Nip and *d61-1* mutant.

**Figure 3:**
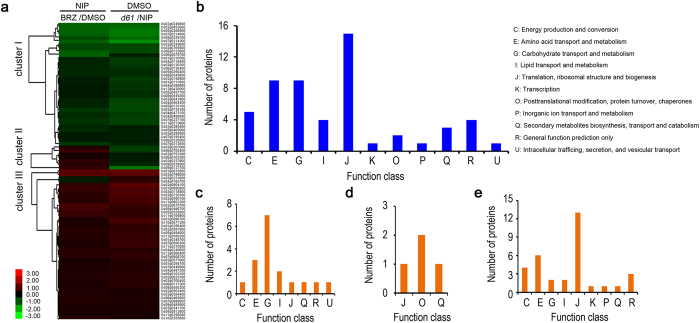
Hierarchical cluster analysis and COG classification of the changed proteins in the embryo of both BRZ treated Nip and *d61-1* mutant. (**a**) Hierarchical cluster analysis of common target proteins in response to both BRZ treatment and OsBRI1 mutation. (**b**) COG classification of the total common changed proteins. (**c**) to (e) COG classification of proteins classified into cluster I, cluster II and cluster III, respectively.

Comparison result showed that total 88 proteins were detected in both two sets of experiments (Fig. 2, Table 1, 2 and 3), which may represent the most creditable BR-regulated proteins involved in rice seed germination. What noteworthy is the fact that 90% of the overlapped proteins exhibited a same abundance change pattern in response to BRZ treatment or *OsBRI1* mutation, with 37 proteins down-regulated (Table 1) and 42 proteins up-regulated (Table 2). Moreover, average fold-changes of down-regulated and up-regulated proteins in BRZ-treated samples were 0.67 and 1.39, respectively, while those in *d61* mutant were 0.61 and 1.50. This result suggested that the set of log2 ratios in *d61* mutant appeared to have slightly wider distribution than that in BRZ-treated NIP, suggesting that BR receptor mutation caused a more striking abundance change of proteins than those caused by BRZ treatment, which was consistent with the fact that more proteins with expression changed were identified in *d61-1* mutant (608 vs 232). These results further consolidated the notion that BR pathway indeed played important roles in seed germination by modulating expression of a large number of target proteins. Therefore, the 88 overlapped target proteins responsive to both BR deficiency and insensitivity were important candidate proteins and were selected for further analysis. First, hierarchical clustering was performed to acquire a comprehensive overview of expression dynamics of these proteins, in which proteins with similar expression change patterns were grouped together. Based on the hierarchical clustering result, the 88 proteins could be generally classified into three clusters, cluster I to cluster III (Fig. 3a). Protein abundance in cluster I was down-regulated and that of almost all the proteins in cluster III was up-regulated in both BRZ-treated NIP and *d61-1* mutant comparing to that of the mock-treated NIP control. While the number of proteins classified into cluster II was only 7 and their expression increased in BRZ-treated embryos and decreased in *d61-1* embryos. Moreover, the 88 proteins were clustered into 11 functional categories by Cluster of Orthologous Groups (COG) assay (Fig. 3b). 15 proteins, accounting for 17% of the 88 proteins, were clustered into the ‘Translation, ribosomal structure and biogenesis’ COG category. Following are the ‘Amino acid transport and metabolism’ and ‘Carbohydrate transport and metabolism’ COG categories with 9 proteins in each category. To get further insight into the protein functions in each cluster, especially cluster I and cluster III, corresponding to the down-regulated and up-regulated BR-responsive proteins, COG analysis were performed to each cluster (Fig. 3c–e). The result showed that ‘Carbohydrate transport and metabolism’ was the major COG category of cluster I (Fig. 3c) while ‘Translation, ribosomal structure and biogenesis’ was the major COG category of cluster III (Fig. 3e), suggesting that BR deficiency and insensitivity led to suppression of starch metabolism process and promotion of biosynthesis of ribosome structure proteins during the early stage of seed germination. Previous reports indicated that starch degraded in the endosperm and the carbohydrates transported into embryo during seed germination, then the carbohydrates were repolymerized into starch granule again^28^,^29^. Re-synthesis of starch in embryo during seed germination might be an effective way for carbohydrate usage and energy provision^30^. Therefore, BR deficiency or insensitivity induced inhibition of starch biosynthesis in the embryo may correlate to the delaying of seed germination and post-germination growth.

**Figure 4:**
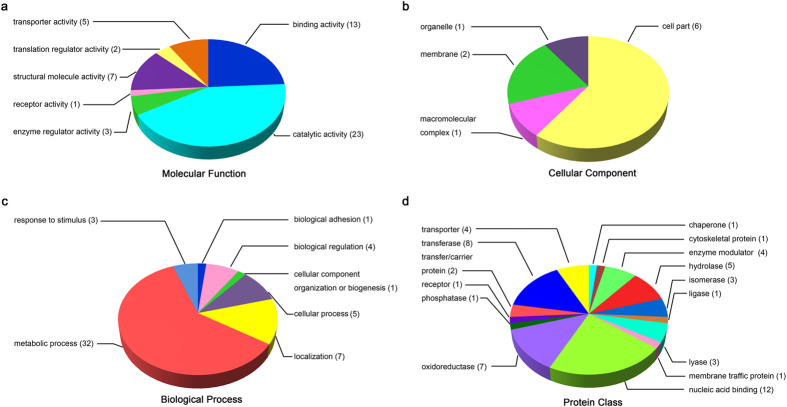
Functional categorization of BR-responsive proteins in the embryos of germinated seeds by using Gene Ontology (GO) analysis. BR-responsive proteins were presented in molecular function (**a**), cellular component (**b**), biological process (**c**) and protein class (**d**), respectively.

To gain knowledge of BR-responsive proteins involved in seed germination, the co-regulated target proteins identified from iTRAQ-based proteomic assay were analyzed separately in the Panther database with four sets of ontologies: molecular function, cellular component, biological process and protein class. It should be mentioned that since some proteins may have no GO annotations in the database, the total number of proteins in each pie chart may less than that of the input. Based on the GO analysis of molecular function, all the proteins could be divided into 7 functional groups, which were catalytic activity, binding activity, transporter activity, translation regulator activity, structural molecule activity, receptor activity and enzyme regulator activity (Fig. 4a). Among them, the catalytic activity group was the largest and the binding activity group was the second largest. As for the cellular component categories, only 4 categories were included and cell part group was the largest one (Fig. 4b). According to the GO analysis of biological process, also 7 functional groups were identified, including metabolic process, response to stimulus, biological adhesion, biological regulation, cellular component organization or biogenesis, cellular process and localization. Specifically, metabolic process group was the largest, corresponding to about 60% of the total identified proteins (Fig. 4c), which was consistent with the fact that during the phase II or the plateau phase of seed germination, reactivation of metabolisms and regulation of genes expression occurring comprehensively, including starch/carbohydrate metabolism, amino acid metabolism^24^. The GO analysis of protein class indicated that the target proteins were classified into 15 groups (Fig. 4d) and the distribution was more even, among which nucleic acid binding made the largest portion (22.2%). This group of proteins, including DNA binding proteins and RNA binding proteins, mainly has function in ATP synthase, DNA methylation, protein translation initiation and elongation, assembling of ribosome proteins, all of which are critical processes during seed germination. Followed nucleic acid binding group was transferase (14.8%), oxidoreductase (13.0%) and hydrolase (9.3%), respectively. These BR-responsive proteins distributed widely and were classified into different functional or cellular categories according to various GO assays. Therefore, the proteomic data supports the notion that BR played an important role in promoting seed germination via modulating various biological processes.

**Figure 5:**
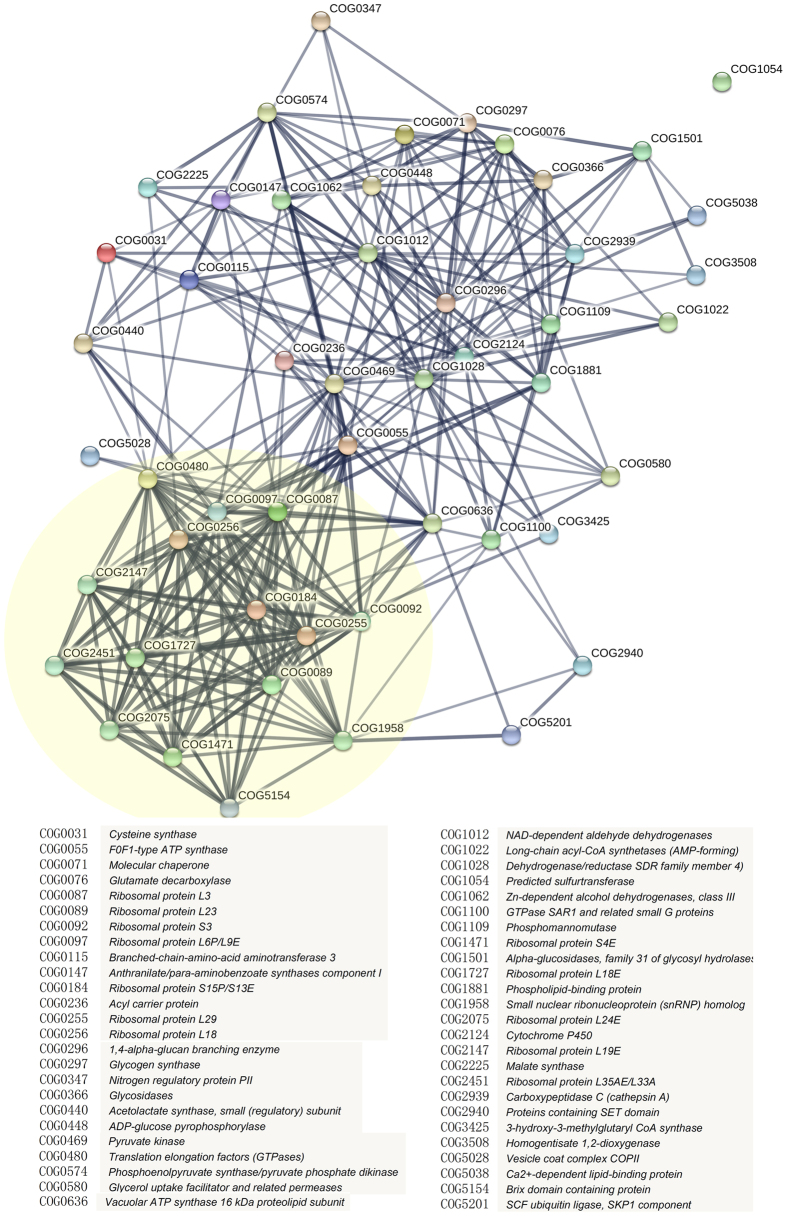
STRING analysis uncovering protein-protein interaction networks of BR-responsive proteins in the embryos of germinated seeds. The proteins within the yellow circle represent the highly clustered ribosomal proteins.

To further clarify the inter-connections among the confidential target proteins, a STRING (Search Tool for the Retrieval of Interacting Genes/Proteins) analysis was performed by using STRING database version 10.0^31^ and it revealed a complex protein association network, in which almost all tested proteins had certain direct or indirect interactions with other proteins. Especially a number of ribosomal proteins were highly clustered (Fig. 5). Moreover, some proteins associated with protein biosynthesis, carbohydrate metabolism, were also among the intensive interaction networks, indicating that BR may regulate seed germination by modulating these biological processes.

### Proteins sensitively regulated by BR pathway

After above general analysis of the proteomic data, we selected 8 proteins with distinct expression change (≥2 fold) in both *d61-1* mutant and BRZ-treated samples for further detailed analysis. Among them, three were glutelin proteins. Specifically, protein abundances of all three glutelin proteins were decreased by more than 50% in the embryos. Just like glutelins, accumulations of RAG2 and RA17, two seed allergenic proteins, were also remarkably declined in BR deficient or BR insensitivity conditions. Moreover, iTRAQ also detected a sharply expression decline of a putative serine carboxypeptidase homologue *OsSCP28*. It is well-known that serine carboxypeptidases are a large family of protein hydrolyzing enzymes that function in a series of biological events, including protein turnover, wound responses, xenobiotic metabolism and seed germination^32^. In comparison to the down-regulated proteins, the number of up-regulated proteins was small with only 2 proteins included. One is Histone-like protein and the other is 60S ribosomal protein L24-A. In consideration of the fact that 12 more ribosomal proteins were also included in the up-regulated protein group (Table 2), it is likely that BR could affect protein biosynthesis via modulating accumulation of ribosomal proteins and thus regulating seed germination process.

### Validation of several BR-responsive genes by qRT-PCR

**Figure 6:**
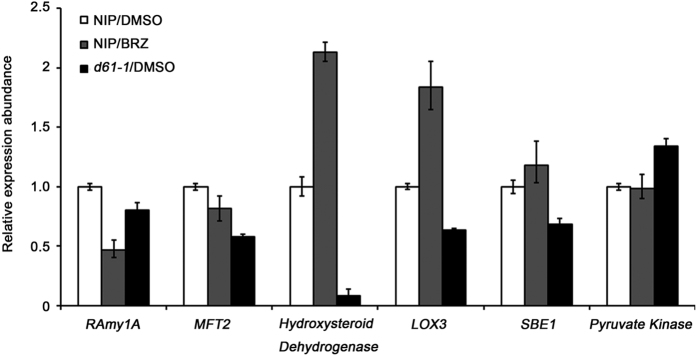
Validation of the proteomic data by analyzing the transcription changes of several selected target genes. Total RNAs were extracted from embryos of rice germinated in darkness for 36 hours. *UBC* was used as the internal control. Each value is the mean of three biological replicates.

Quantitative RT-PCR analysis is a widely adopted method to validate proteomic results. Thus, we selected several representative common target genes for qRT-PCR validation. First, four GA or ABA responsive genes were chosen, including *RAmy1A*, *OsMFT2*, *Hydroxysteroid Dehydrogenase* and *LOX3*. Since it is well-known that GA and ABA played a major role in seed germination, it is important to clarify whether BR modulates seed germination via co-regulate common target genes with GA and ABA. Second, *SBEI* and *Pyruvate Kinase*, two common target genes involved in starch biosynthesis or glucose breakdown, were also selected because sugar metabolism is a critical biological process during rice seed germination. Therefore, total six target genes were selected for further qRT-PCR validation. Generally, the transcription change of *RAmy1A* and *OsMFT2* was consistent with the proteomic results, declined in both BR-deficient and BR-insensitive seeds (Fig. 6). However, the other four genes exhibited a different transcription change in the two sets of materials (Fig. 6). In fact, proteomic data showed that all the six target genes had a similar protein abundance change pattern in response to both BR-deficient and BR-insensitive stimuli. It was also interesting to note that 5 out of 6 target genes shared a similar expression change in both transcriptional and translational levels in mutant *d61-1*. While in the BRZ-treated samples only three target genes had such a consistent expression change.

## Discussion

Successful transformation of a tiny seed into a normal seedling accompanied drastic morphological changes. Seed germination is a decisive event for the next-generation plant growth and successful seed germination is critical for crop yield. Due to the rich genetic resources, mechanism of seed germination has been well-studied in Arabidopsis. A number of target genes correlated with altered seed dormancy or germination have been identified and isolated, many of which were involved in GA and ABA biosynthesis or signaling^30^,^33^,^34^. However, molecular mechanisms of seed germination in crop species, such as rice, are still largely unclear^35^. Thus, understanding the molecular mechanisms of rice seed germination will benefit rice production and new hybrid cultivation.

Rice seed germination is regulated by a series of environmental and internal stimuli, including plant hormones^24^,^36^. Recently, results from RNA-seq expression profile of rice embryos revealed the phytohormone interaction network during seed germination and six of the top ten hub genes belonged to ABA, GA and BR pathways^37^, further highlighted the distinct roles of phytohormones, including BR, in rice seed germination. Although a number of studies in rice have successfully revealed how BR regulates stem elongation, grain size^3^,^25^,^38^,^39^, mechanisms on how BR modulates seed germination is still unclear. In this study, we investigated BR-responsive proteins by using iTRAQ approach and totally identified 840 proteins, 88 of which were co-regulated by both BR deficiency and BR insensitivity.

*d61-1* is a mild *OsBRI1* mutant with less sensitivity to BRs comparing to wild-type plants^25^. Although we identified around 600 proteins with expression changed in *d61-1*, 92.3% proteins had less than 2-fold expression changes. It is well-known that BR signaling pathway could modulate BR biosynthesis in a type of feedback regulation^40^. For instance, a number of identified *d61* alleles were classified into three groups according to plant height and fertility, among which *d61-1*, *d61-2* and *d61-4* belonged to the mild, intermediate and severe groups, respectively^41^. Quantitative analysis of BRs indicated that about 4-fold and 30-fold of bioactive cathasterones were detected in *d61-2* and *d61-4*, respectively^25^,^41^, suggesting that endogenous BR content increased in the BR-insensitive mutants and closely correlated with severity of mutant phenotypes. Therefore, BR signaling mutants could compensate loss of BRI1 activity to certain extent by increasing endogenous BRs, which may be one of the reasons leading to the relative mild expression changes of detected proteins in *d61-1*. To eliminate this effect, BRZ could be used to block endogenous BR biosynthesis in BR insensitive mutants in future research. In this way, more proteins with strikingly expression changes should be identified. Another possibility is that fold changes of protein abundance from iTRAQ assay were easily underestimated, which phenomena was also reported by some other iTRAQ researches in plants^42^,^43^. In this study, only 172 and 50 proteins with expression changed could be isolated from *d61* mutant and BRZ-treated NIP with a 1.5-fold-change criterion, among which, only 18 proteins belonged to the overlapped common target proteins (Supplemental Table S5). Furthermore, protein numbers identified in mutant *d61-1* was bigger than that in the BRZ-treated wild-type samples. It is possible that although BRZ-treatment could mimic the BR-deficient conditions, 36-hour treatment could not absolutely exhaust the endogenous BRs. Therefore, a certain amount of endogenous BRs may still exist and have functions during seed germination, which might cause a relative mild effect on rice seed germination and result in a smaller number of changed proteins.

Although great progress has been made in identifying BR-regulated genes in the transcriptional level^13^,^15^, studies on dissecting BR-responsive proteins are relatively rare. Previous two proteomic studies in Arabidopsis and rice only identified 42 and 36 BR-responsive proteins, respectively^44^,^45^. However, BR-regulated proteomic study on rice seed, the most important organ of rice, has not been reported yet. Our iTRAQ study identified more than 800 BR-responsive proteins, among which 88 proteins were common targets of both BR-deficiency and BR-insensitivity. Moreover, 22 of the 88 BR-responsive proteins were exclusively or preferentially expressed in seeds by referring to the SeedGeneDB databse (http://sgdb.cbi.pku.edu.cn/). Furthermore, 90% of the overlapped BR-responsive proteins have consistent expression change patterns in both BR-deficient and BR-insensitive rice embryos, suggesting that these proteins might mediate BR-modulated rice seed germination. COG classification of the common target proteins indicated that protein-biosynthesis-related proteins were highly clustered (Fig. 3b). Recently, two-dimensional electrophoresis (2-DE) approaches were performed to identify proteins involved in early stage of rice seed germination. Results showed that proteins related to metabolism and protein biosynthesis made up a large proportion of the identified proteins^46^,^47^. A phosphoproteomic study also emphasized crucial roles of protein-synthesis-related proteins in the early stage of rice seed germination, during which 83% of the identified nuclear phosphoproteins related to protein synthesis^48^. Further GO analysis showed that protein ratios with catalytic activities and involved in the metabolism process was high (Fig. 4a–d), which is consistent with that the major event in phase II of seed germination is reactivation of metabolism^28^. Therefore, expression alteration of proteins related to metabolism would notably affect seed germination. For instance, ABA treatment or high temperature notably modulated expression of enzymes involved in energy metabolism and protein synthesis, thus leading to a reduced seed germination of rice^49^.

Protein association network analysis by STRING database further highlighted the critical roles of proteins involved in protein biosynthesis during rice seed germination, especially ribosomal proteins. Protein abundance of almost all the identified ribosomal proteins increased in response to stimulus of BR-deficiency or BR-insensitivity. In fact, a previous proteomic analysis of rice embryo also identified a number of ribosomal proteins with expression changed under normal germination conditions, in which 26 out of 30 proteins with expression decreased^29^. These results indicated that accumulation of ribosomal proteins is closely correlated with seed germination. Therefore, BR-regulated ribosomal protein abundance may play an important role in orchestrate seed germination process. Besides seed germination, proteomic studies indicated that protein-synthesis-related proteins and ribosomal proteins also played important roles in forming rice chalky grains by modulating energy and metabolic pathways^50^,^51^. Recently, study also showed that BR involved in the energy metabolism regulation under stress conditions by inducing de novo protein synthesis^52^.

In addition, our qRT-PCR validation result indicated that four out of six tested genes only shared a modest correlation between the transcription and translation results, implying that although qRT-PCR is a widely used method to confirm proteomic results in transcriptional level, transcription and translation changes are not always consistent. In fact, a number of studies reported inconsistent results between proteomic and transcriptomic data^53^,^54^. Recent two studies further highlighted this notion. Peng *et al.*^55^ integrated proteome and transcriptome methods to study gene regulation network in *dl2* mutant in rice^55^. Proteome and transcriptome data indicated that 141 proteins and 98 mRNAs were differentially expressed between *dl2* mutant and its wild-type control. However, only two genes overlapped between the proteome and transcriptome, suggesting only a weak correlation between protein and transcript levels. A similar result was also reported by Li *et al.*^56^, who studied rice pistil response during early post-pollination via a joined proteomic and transcriptomic analysis^56^. Totally 962 transcripts and 167 proteins were identified with differential expression. However, only 12 genes showed expression changes at both RNA and protein levels. The above comparison results further reinforced the notion that in most cases notable deviation existed between results of proteome and transcriptome.

Due to improvement of sensitivity and accuracy of iTRAQ method, we totally identified 840 proteins in response to BR deficiency or BR insensitivity in the present study, which was larger than an earlier report by using 2-DE method^44^. Furthermore, what we chose for experiment was embryos from germinated rice seeds, which determines whether the seed could germinate successfully and begin a new generation of life-cycle. It was quite interesting to note that 9 out of the 36 proteins from Wang *et al.*^44^ were also detected in our iTRAQ data. Since these 9 proteins were from different plant tissues, including shoots and roots of seedlings as well as embryos from germinated seeds, they should be important and reliable BR-responsive targets with a wide spectrum of functions. In detail, the proteins included two methionine synthase and one methionine sulfoxide reductase. Methionine, a fundamental metabolite, functions not only as a building block for protein biosynthesis but also as the precursor of S-adenosylmethionine (SAM) and polyamines^57^. Presence of methionine synthesis inhibitor strongly delayed Arabidopsis seed germination, suggesting methionine biosynthesis is essential for Arabidopsis seed germination^58^. Moreover, Liu *et al.*^49^ reported that abundance of proteins involved in methionine metabolism decreased in response to both high temperature and ABA treatment inhibited seed germination^49^. Therefore, modulation of methionine metabolism might be an effective way for BR to regulate rice seed germination. In addition, a fructokinase and a fructose-bisphosfate aldolase were also included, both functioning in fructose metabolism. Fructokinase were also involved in vascular development, starch biosynthesis, and fructose sensing^59^, while fructose-bisphosfate aldolase has functions in stress resistance^60^. Another important protein is DREPP, a known BR-induced plasma membrane protein, which functions as a positive regulator of cell expansion in both rice and Arabidopsis^44^,^61^. Finally, a GTP binding protein, a Thioredoxin peroxidase and an Actin protein were also among the 9 overlapped BR-responsive proteins.

In summary, our iTRAQ study has disclosed more than 800 BR-responsive proteins involved in rice seed germination, including 88 proteins with high confidence. Moreover, about 90% of the 88 common target proteins shared a similar expression change pattern in response to stimuli of both BR-deficiency and BR insensitivity. Gene ontology and String analysis indicated that many proteins involved in protein biosynthesis, carbohydrate metabolism and other biological processes were highly clustered. Therefore, our data set not only enriches BR-regulated protein database, but also allows us to gain novel insights into the molecular mechanism of BR regulated seed germination.

## Methods

### Seed germination

Both *d61-1* mutant and its wild-type control Nipponbare (*Oryza sativa* L. ssp *japonica*) were grown in the same growth conditions and their mature seeds were collected for germination experiments. Three biological replicates were used and each replicate contained 100 seeds. Briefly, rice seeds were manually dehulled, washed once with 70% ethanol and following twice wash by Milli-Q grade water. Then seeds were imbibed with a certain amount of Milli-Q grade water supplemented with 1 μM BRZ or mock in darkness at 26 °C. After germination for 36 hours, embryos were separated from seeds and quickly froze by liquid nitrogen. Then the frozen embryos were stored in −80 °C until used for protein extraction. For seed weight analysis, three replicates, each containing 30 seeds, were used with the same treatment and growth conditions. The data of seed weight were collected after germination for 0, 12, 24, 36, 48, 60 and 72 hours.

### Protein extraction

Rice embryo samples from three biological replicates were mixed and ground into powder in liquid nitrogen, extracted with lysis buffer (7 M Urea, 2 M Thiourea, 4% CHAPS, 40 mM Tris-HCl, pH 8.5, 1 mM PMSF and 2 mM EDTA). After 5 min, 10 mM DTT (final concentration) was added to the samples. The suspension was sonicated at 200 W for 15 min and then centrifuged for another 15 min (30000 g, 4 °C). The supernatant was mixed well with 5 × volume of chilled acetone containing 10% (v/v) TCA and incubated at −20 °C overnight. Then the supernatant was discarded after centrifugation at 4 °C, 30 000 g. Next the precipitate was washed three times with chilled acetone and the pellet was air-dried and dissolved in Lysis buffer (7M urea, 2 M thiourea, 4% NP40, 20 mM Tris-HCl, pH 8.0–8.5). Again, the suspension was sonicated (200 W, 15 min) and centrifuged (15 min, 4 °C, 30000 g) and the supernatant was transferred to a new tube. Then 10 mM DTT was added to reduce disulfide bonds in proteins by incubating at 56 °C for 1 h. Subsequently, 55 mM IAM was added and incubated for 1 h in darkness to block the cysteines. The supernatant was mixed well with chilled acetone (5 × volume, 2 h, −20 °C) to precipitate proteins. After centrifugation (30000 g, 4 °C), the pellet was got and air-dried for 5 min, and then dissolved in 500 μL TEAB (0.5 M, Applied Biosystems, Milan, Italy) and followed by a 15 min sonication (200 W). Finally, samples were centrifuged (15 min, 4 °C, 30000 g) and the supernatant was collected and kept at −80 °C for further analysis.

### iTRAQ Labeling

100 μg total proteins were digested (37 °C, 16 hours) with Gold grade Trypsin (Promega, Madison, WI, USA) with the ratio of 30:1 (protein: trypsin). Then the peptides were dried by vacuum centrifugation and reconstituted in 0.5 M TEAB and labeled with 8-plex iTRAQ reagent (Applied Biosystems). The labeled peptide mixtures were incubated at room temperature for 2 h and dried by vacuum centrifugation, and then followed by SCX fractionation with a LC-20AB HPLC Pump system (Shimadzu, Kyoto, Japan). Finally, the peptides were pooled into 20 fraction and desalted with a Strata X C18 column (Phenomenex) and vacuum-dried.

### LC-ESI-MS/MS analysis

Each fraction was resuspended in buffer A (5% ACN, 0.1%FA) and centrifuged at 20000g for 10 min. 10 μl supernatant was loaded on a LC-20AD nano-HPLC (Shimadzu, Kyoto, Japan) and the peptides were eluted onto a 10 cm analytical C18 column (inner diameter 75 μm) for separation. The data were acquired by using a TripleTOF 5600 System (AB SCIEX, Concord, ON), fitted with a Nanospray III source (AB SCIEX, Concord, ON) and a pulled quartz tip as the emitter (New Objectives, Woburn, MA). Data was acquired using a 2.5 kV ion spray voltage, 30 psi curtain gas, 15 psi nebulizer gas, and 150 °C interface heater temperature. The resolving power for TOF MS scans was greater than 30000 FWHM. For IDA, survey scans were acquired in 250 ms and total cycle time was fixed to 3.3 s. Q2 transmission window was 100Da for 100%. By monitoring the multichannel TDC detector with four-anode channel detection, four time bins were summed for each scan at a pulser frequency value of 11 kHz. A sweeping collision energy (35 ± 5 eV) and iTRAQ with rolling collision energy adjusted was applied to all precursor ions for collision-induced dissociation. Dynamic exclusion was set for 1/2 of peak width (15 s), and then the precursor was refreshed off the exclusion list.

### Proteomic data analysis

Raw data files acquired from the Orbitrap were converted into MGF files using Proteome Discoverer 1.2 (PD 1.2, Thermo), and the MGF file were searched. Protein identification was performed by using Mascot search engine (Matrix Science, London, UK; version 2.3.02) against database containing rice sequences. The charge states of peptides were set to +2 and +3. Specifically, an automatic decoy database search was performed in Mascot by choosing the decoy checkbox in which a random sequence of database is generated and tested for raw spectra as well as the real database. To reduce the probability of false peptide identification, only peptides at the 95% confidence interval by a Mascot probability analysis greater than “identity” were counted as identified. And each confident protein identification involves at least one unique peptide. For protein quantitation, it was required that a protein contains at least two unique peptides. The quantitative protein ratios were weighted and normalized by the median ratio in Mascot. Therefore, the proteins with p-values <0.05, and fold-change ratios ≥1.2 or ≤0.83 were considered as significant.

### Bioinformatic analysis

Hierarchical clustering of protein expression patterns was performed using Cluster software version 3.0 and results were visualized by Treeview software^62^. Functional annotations of the proteins were conducted using Blast2GO program against the non-redundant protein database (NR;NCBI). The Cluster of Orthologous Groups of proteins (COG) was used to classify and group the identified proteins. COG is the database for protein orthologous classification (http://www.ncbi.nlm.nih.gov/COG/). Every protein in COG is supposed to derive from a same protein ancestor. Gene Ontology (GO) is an international standardization of gene function classification system, which provides a set of dynamic updating controlled vocabulary to describe genes and gene products attributes in the organism. The proteins were analyzed separately against the ontologies of GO in the PANTHER database with four sets of ontologies: molecular function, cellular component, biological process and protein class^63^. Protein-protein interaction networks (interactome) of differentially expressed proteins were constructed using STRING 10.0 (Search Tool for the Retrieval of Interacting Genes/Proteins). The name of each individual protein was given as a query to the STRING database and the corresponding PPI information were retrieved by enabling different prediction methods, such as textmining, experiments, databases, co-expression, neighborhood, gene fusion and co-occurrence^31^. The networks were made with a confidence cutoff of 0.6.

### RNA isolation and quantitative real-time PCR (qRT-PCR) analysis

Total RNA was extracted from the seperated embryos from germinated seeds by using the RNeasy plant mini kit (Qiagen), then treated with DNase I (Qiagen) and reverse transcribed by using the SuperScriptTM first-strand synthesis system (Invitrogen). Then qRT-PCR was performed by using the SYBR Premix Ex TaqTM II system (TaKaRa) and the iQ SYBR-Green Supermix (Bio-Rad). Ubiquitin Conjugase (UBC) was used as the internal control for BR-related and light-related experiments, respectively. Primer sequences for target genes were listed in the Supplemental Table S6.

## Additional Information

**How to cite this article**: Li, Q.-F. *et al.* Dissection of brassinosteroid-regulated proteins in rice embryos during germination by quantitative proteomics. *Sci. Rep.* **6**, 34583; doi: 10.1038/srep34583 (2016).

## Supplementary Material

Supplementary Information

## Figures and Tables

**Table 1:** List of proteins down-regulated in embryos of germinated rice seeds in response to BR-deficiency and BR-insensitivity by iTRAQ.

Gene ID	NIP(BRZ/Mock) fold change	d61/NIP fold change	Protein score	Unique Peptide	Sequence coverage(%)	Description
Os01g0111600	0.793	0.653	230	3	20.7	Similar to MOTHER of FT and TF1 protein.
Os01g0210500	0.762	0.633	1133	6	45	Similar to SOUL-like protein.
Os02g0158900	0.816	0.626	244	6	18.8	Similar to SNF4.
Os02g0249000	0.406	0.411	804	10	22.7	Glutelin, Seed strage protein
Os02g0249600	0.568	0.57	3780	1	34.9	Similar to Glutelin.
Os02g0249800	0.452	0.45	3900	2	36.3	Glutelin precursor. >Os02t0249900-01 Glutelin precursor.
Os02g0453600	0.459	0.414	539	5	28.1	Similar to Glutelin.
Os02g0765600	0.576	0.561	660	9	32.5	Alpha-amylase glycoprotein, Degradation of starch granule
Os03g0130300	0.742	0.656	122	2	27.2	Similar to Cp-thionin.
Os03g0295800	0.803	0.78	634	2	10.9	lysosomal thiol reductase GILT family protein.
Os03g0385400	0.725	0.781	882	4	30.3	Bifunctional inhibitor/plant lipid transfer protein
Os03g0734200	0.639	0.566	254	1	17.4	Conserved hypothetical protein.
Os03g0842900	0.768	0.592	1047	13	43.9	Similar to Steroleosin-B.
Os04g0118400	0.75	0.628	7065	1	51.2	Similar to Elongation factor EF-2.
Os04g0390800	0.818	0.669	2520	16	54.8	NAD(P)-binding domain containing protein.
Os04g0404400	0.753	0.577	841	7	31.1	Similar to H0502B11.4 protein.
Os04g0473150	0.7	0.604	703	2	22.2	Similar to photosystem II protein D
Os04g0486950	0.717	0.608	721	11	26.3	Similar to Malate synthase.
Os05g0133100	0.757	0.571	229	2	9.4	Similar to PII protein.
Os05g0268500	0.451	0.369	131	5	12	Similar to Serine carboxypeptidase 2.
Os05g0329100	0.555	0.421	917	3	42.7	Prolamin.
Os05g0358400	0.729	0.715	115	3	11.4	Butirosin biosynthesis, BtrG-like domain containing protein.
Os05g0405000	0.718	0.783	1494	14	22.5	Orthophosphate dikinase precursor.
Os05g0457700	0.806	0.705	186	4	15.9	Tetratricopeptide-like helical domain containing protein.
Os06g0133000	0.482	0.57	495	9	22.2	Granule-bound starch synthase I, chloroplast precursor
Os06g0320000	0.8	0.8	127	3	30.2	Thioredoxin fold domain containing protein.
Os06g0675700	0.463	0.781	831	4	18	Similar to High pI alpha-glucosidase
Os06g0726400	0.743	0.678	591	12	15.7	Branching enzyme-I precursor (Starch-branching enzyme I)
Os07g0195400	0.799	0.771	496	9	23.7	Phosphoacetylglucosamine mutase domain-protein.
Os07g0213600	0.627	0.747	238	2	24.7	Bifunctional inhibitor/plant lipid transfer protein
Os07g0214300	0.474	0.321	1815	2	28.3	Seed allergenic protein RAG2 precursor.
Os07g0214600	0.465	0.383	606	3	25.5	Similar to Seed allergenic protein RA17 precursor.
Os07g0237100	0.667	0.59	121	2	8.5	RNA recognition motif domain domain containing protein.
Os08g0345800	0.729	0.695	289	5	22.5	AGPS2;Glucose-1-phosphate adenylyltransferase small subunit
Os08g0549300	0.822	0.687	403	1	10.9	Similar to Acyl carrier protein III (ACP III).
Os11g0213600	0.682	0.6	180	5	11.6	Peptidase S10, serine carboxypeptidase family protein.
Os12g0430000	0.815	0.662	414	5	10.2	Hypothetical conserved gene.

**Table 2:** List of proteins up-regulated in embryos of germinated rice seeds in response to BR-deficiency and BR-insensitivity by iTRAQ.

Gene ID	NIP(BRZ/Mock) fold change	d61/NIP fold change	Protein score	Unique Peptide	Sequence coverage(%)	Description
Os01g0294700	1.239	1.317	707	8	33.7	Haem peroxidase, plant/fungal/bacterial family protein.
Os01g0348700	1.319	1.552	422	3	20.4	Similar to 60S ribosomal protein L23a (L25).
Os01g0358400	1.259	1.475	2745	1	43.4	Similar to 40S ribosomal protein S4.
Os01g0815800	2.061	2.224	682	1	19.8	Similar to 60S ribosomal protein L24-A (L30A) (RP29).
Os01g0880800	1.585	1.835	891	5	16.8	Similar to Acyl-[acyl-carrier-protein] desaturase
Os01g0896700	1.268	1.701	902	3	34.5	Similar to 60S ribosomal protein L5.
Os02g0503400	1.29	1.229	99	2	13.8	Similar to 60S ribosomal protein L35.
Os02g0550100	1.38	1.726	419	1	10.8	Similar to Vacuolar ATP synthase 16 kDa proteolipid
Os02g0591800	1.347	1.451	86	3	13	Brix domain containing protein.
Os02g0675700	1.741	1.617	236	4	18.4	methyltransferase putative family protein.
Os02g0804100	1.486	2.092	242	1	16.1	Similar to predicted protein.
Os03g0118800	1.486	1.8	176	3	12.7	Similar to Hydroxymethylglutaryl-CoA synthase.
Os03g0264400	1.317	1.225	186	4	11.2	Anthranilate synthase alpha 2 subunit.
Os03g0341100	1.234	1.265	506	4	28.3	Similar to 60S ribosomal protein L18.
Os03g0577000	1.222	1.377	1613	6	45.6	Similar to Ribosomal protein S3.
Os03g0700400	1.297	1.399	1056	3	37.3	Similar to LOX4.
Os03g0720300	1.384	1.884	452	5	26.6	Similar to Glutamate decarboxylase isozyme 1.
Os03g0799000	1.997	2.018	126	2	18.4	Similar to Histone H1.
Os03g0823700	1.307	1.421	564	3	40.9	Similar to Ras-related protein Rab11C.
Os04g0249600	1.371	1.389	151	2	23.9	Rhodanese-like domain containing protein.
Os04g0497200	1.275	1.348	169	4	9.3	Cellulase precursor (Cellulase homolog OR16pep).
Os05g0103100	1.298	1.36	788	3	23.1	Translocon-associated beta family protein.
Os05g0486700	1.676	1.457	509	1	19.9	Ribosomal protein L24e domain containing protein.
Os05g0494000	1.334	1.492	231	6	13.9	Similar to Cytochrome P450 98A1.
Os05g0512600	1.221	1.224	365	2	15.4	X8 domain containing protein.
Os05g0555800	1.545	1.474	290	2	37.5	Similar to 60S ribosomal protein L35a-3.
Os07g0448800	1.255	1.309	531	5	18.3	Aquaporin.
Os07g0500300	1.293	1.563	303	3	32.4	C2 calcium-dependent membrane targeting domain protein.
Os07g0608700	1.423	1.474	124	2	32.5	Similar to small nuclear ribonucleoprotein G.
Os07g0688800	1.321	1.266	248	4	8.5	Aldehyde dehydrogenase domain containing protein.
Os08g0117300	1.283	1.39	2212	3	57	Similar to 40S ribosomal protein S13.
Os08g0465800	1.304	1.291	952	8	33.4	Similar to Glutamate decarboxylase.
Os08g0555200	1.298	1.381	238	3	6.7	Nonaspanin (TM9SF) family protein.
Os09g0485900	1.294	1.256	1741	1	41.1	Similar to 60S ribosomal protein L9.
Os10g0355800	1.391	1.221	2143	4	38.1	Similar to ATP synthase CF1 beta subunit.
Os10g0571200	1.256	1.679	424	6	18.8	Similar to Pyruvate kinase isozyme G, chloroplast.
Os11g0168200	1.322	1.503	1855	11	28	60S ribosomal protein L3.
Os11g0169800	1.576	1.457	100	3	11.3	Similar to Long-chain-fatty-acid–CoA ligase 4.
Os11g0210500	1.25	1.581	1940	6	39.8	Similar to Alcohol dehydrogenase.
Os11g0256050	1.221	1.225	177	4	22.1	Hypothetical conserved gene.
Os11g0602200	1.41	1.744	219	3	6.4	Similar to SET domain protein SDG111.
Os12g0506400	1.387	1.415	95	1	6.7	Cornichon family protein.

**Table 3:** List of proteins inconsistently-regulated in embryos of germinated rice seeds in response to BR-deficiency and BR-insensitivity.

Gene ID	NIP(BRZ/Mock) fold change	d61/NIP fold change	Protein score	Unique Peptide	Sequence coverage(%)	Description
Os02g0753300	1.212	0.826	1514	4	26.9	Lipoxygenase, LH2 domain containing protein.
Os03g0231600	0.799	1.365	241	3	9.4	Similar to Atbcat-3.
Os03g0267000	1.242	0.613	301	2	41	Low molecular mass heat shock protein Oshsp18.0.
Os03g0337800	1.467	0.775	356	2	24	Similar to 60S ribosomal protein L19.
Os04g0165700	0.804	1.467	312	3	9.5	Cysteine synthase.
Os06g0103300	1.203	0.778	370	3	9.1	Similar to Homogentisate 1,2-dioxygenase.
Os06g0705400	1.607	0.609	205	1	11.7	Similar to Nonspecific lipid-transfer protein 2P.
Os09g0127700	1.506	0.4	221	2	21.5	Conserved hypothetical protein.
Os09g0539500	1.367	0.716	784	3	36.6	Similar to SKP1-like protein 1A.
