# Supplementary material for: Dissection of brassinosteroid-regulated proteins in rice embryos during germination by quantitative proteomics
Source: Sci Rep. 2016 Oct 5;6:34583. doi: 10.1038/srep34583 (PMC5050409; doi:10.1038/srep34583)
Supplement: Supplementary Information [file srep34583-s1.pdf]

Supplementary Information

Dissection of brassinosteroid-regulated proteins in rice embryos during germination by quantitative proteomics

Qian-Feng Li, Min Xiong, Peng Xu, Li-Chun Huang, Chang-Quan Zhang, Qiao-Quan Liu*

Supplementary Figure S1 Images of germinating Nipponbare seeds.

Supplementary Figure S2 SDS-PAGE analysis of extracted proteins.

Supplementary Figure S3 Protein ratio distribution analysis.

Supplementary Table S1 List of proteins down-regulated in embryos of germinated rice seeds in response to BRZ-treatment by iTRAQ.

Supplementary Table S2 List of proteins up-regulated in embryos of germinated rice seeds in response to BRZ-treatment by iTRAQ.

Supplementary Table S3 List of proteins down-regulated in embryos of germinated rice seeds of mutant d61-1 by iTRAQ.

Supplementary Table S4 List of proteins up-regulated in embryos of germinated rice seeds of mutant d61-1 by iTRAQ.

Supplementary Table S5 List of common target proteins in embryos of germinated rice seeds in response to both BR-deficiency and BR-insensitivity by iTRAQ (fold-change criterion ≥1.5 or ≤0.67)

Supplementary Table S6 List of primers used for quantitative real-time PCR assay

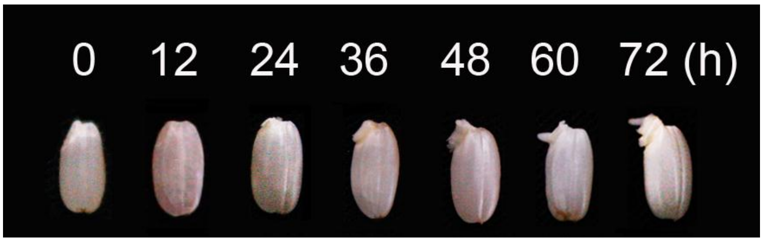

Supplementary Figure S1 Images of germinating Nipponbare seeds.

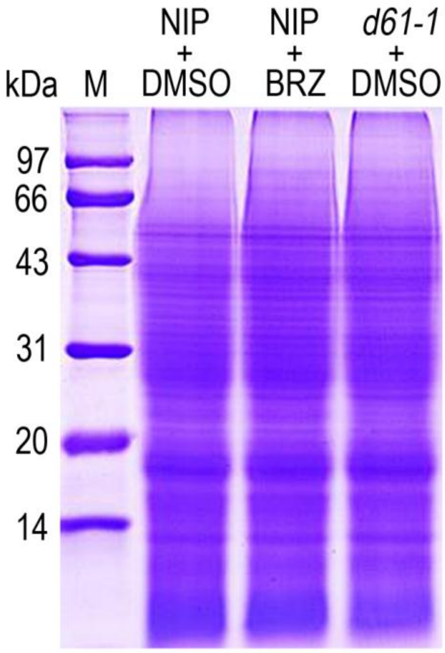

Supplementary Figure S2 SDS-PAGE analysis of extracted proteins.

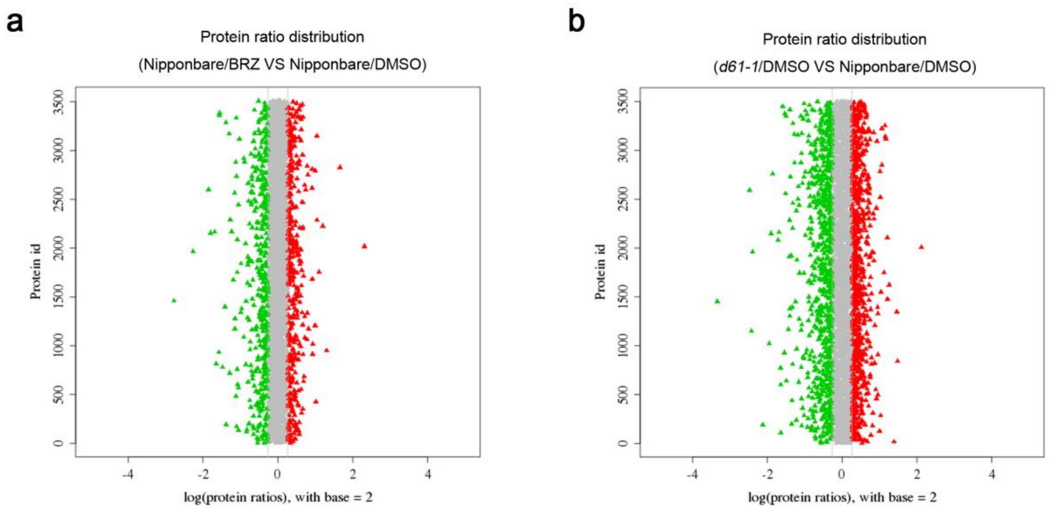

Supplementary Figure S3 Protein ratio distribution analysis.

Supplementary Table 1. List of proteins down-regulated in embryos of germinated rice seeds in response to BRZ-treatment by iTRAQ

Gene ID	NIP(BRZ / Mock) fold change	Protein score	Unique Peptide	Sequence coverage(%)	Description
Os02g0248800	0.208	1443	3	7.1	Similar to Glutelin type-B 2 precursor.
Os03g0427300	0.336	2685	10	30.2	Glutelin type-A III precursor.
Os05g0499100	0.384	757	4	25.8	26 kDa globulin (Alpha-globulin).
Os02g0249000	0.406	804	10	22.7	Glutelin, Seed strage protein
Os06g0507200	0.414	734	4	22.8	Bifunctional inhibitor/plant lipid transfer protein.
Os05g0268500	0.451	131	5	12	Similar to Serine carboxypeptidase 2.
Os02g0249800	0.452	3900	2	36.3	Glutelin precursor. >Os02t0249900-01 Glutelin precursor.
Os02g0453600	0.459	539	5	28.1	Similar to Glutelin.
Os06g0675700	0.463	831	4	18	Similar to High pI alpha-glucosidase.
Os08g0530400	0.465	71	2	8.8	Moco containing protein (Moco containing protein(OsMCP)).
Os07g0214600	0.465	606	3	25.5	Similar to Seed allergenic protein RA17 precursor.
Os07g0214300	0.474	1815	2	28.3	Seed allergenic protein RAG2 precursor.
Os06g0133000	0.482	495	9	22.2	Granule-bound starch synthase I, chloroplast precursor
Os07g0214100	0.506	344	3	28.2	Seed allergenic protein RA17 precursor.
Os03g0760800	0.532	166	2	29	Similar to GAST1 protein precursor.
Os05g0329100	0.555	917	3	42.7	Prolamin.
Os02g0249600	0.57	3780	1	34.9	Similar to Glutelin.
Os02g0765600	0.576	660	9	32.5	Alpha-amylase glycoprotein, Degradation of starch granule
Os03g0812000	0.587	305	2	6.6	DNA topoisomerase, type IIA,
Os06g0676700	0.593	981	5	18.4	Similar to High pI alpha-glucosidase.
Os07g0574800	0.622	3113	3	56.7	Tubulin alpha-1 chain. >Os07t0574800-02 Tubulin alpha-1 chain.
Os11g0582400	0.626	702	2	5.1	Similar to Embryo-specific protein.

Os06g0214300	0.627	60	2	9.9	Alpha/beta hydrolase fold-3 domain containing protein.
Os07g0213600	0.627	238	2	24.7	Bifunctional inhibitor/plant lipid transfer protein
Os03g0734200	0.639	254	1	17.4	Conserved hypothetical protein.
Os05g0499600	0.65	172	3	10.7	UDP-glucuronosyl/UDP-glucosyltransferase family protein.
Os07g0529600	0.65	333	6	25.1	Similar to Thiazole biosynthetic enzyme 1-1, chloroplast precursor.
Os04g0628600	0.665	251	5	5.5	Conserved hypothetical protein.
Os07g0237100	0.667	121	2	8.5	RNA recognition motif domain domain containing protein.
Os11g0213600	0.682	180	5	11.6	Peptidase S10, serine carboxypeptidase family protein.
Os02g0816500	0.689	110	1	10	Tubulin binding cofactor A family protein.
Os02g0209300	0.69	78	1	13.7	Hypothetical conserved gene.
Os05g0111300	0.692	56	2	26.2	Similar to B22EL8 protein.
Os01g0652800	0.693	133	3	8.9	Protein of unknown function DUF231.
Os04g0676700	0.696	106	2	7.3	Similar to H0101F08.8 protein.
Os07g0301200	0.698	187	4	9.4	Similar to RNA helicase (Fragment).
Os04g0473150	0.7	703	2	22.2	Similar to photosystem II protein D1.
Os06g0473800	0.704	467	2	11.7	Oleosin family protein.
Os04g0486950	0.717	721	11	26.3	Similar to Malate synthase.
Os05g0405000	0.718	1494	14	22.5	Orthophosphate dikinase precursor (EC 2.7.9.1).
Os03g0207250	0.719	131	3	26.1	Ribosomal protein/NADH dehydrogenase domain protein.
Os03g0385400	0.725	882	4	30.3	Bifunctional inhibitor/plant lipid protein.
Os08g0345800	0.729	289	5	22.5	Similar to Glucose-1-phosphate adenylyltransferase small subunit
Os05g0358400	0.729	115	3	11.4	Butirosin biosynthesis, BtrG-like domain containing protein.
Os06g0139900	0.73	262	1	26.4	Similar to Beta 1 subunit of 20S proteasome.
Os12g0543600	0.738	72	2	6.4	Similar to sarcosine oxidase.
Os02g0139700	0.739	232	7	9.7	Similar to Cycloartenol synthase.
Os06g0708832	0.742	81	4	14.4	Similar to arogenate dehydrogenase.

Os03g0130300	0.742	122	2	27.2	Similar to Cp-thionin.
Os05g0104650	0.743	338	9	11.1	Similar to Formylglycinamide ribonucleotide amidotransferase.
Os06g0726400	0.743	591	12	15.7	Branching enzyme-I precursor (Starch-branching enzyme I).
Os11g0636900	0.748	181	4	8.9	Nucleotide-binding, alpha-beta plait domain containing protein.
Os04g0118400	0.75	7065	1	51.2	Similar to Elongation factor EF-2 (Fragment).
Os03g0798600	0.752	167	1	12.3	Similar to 40S ribosomal protein S15 (Fragment).
Os04g0404400	0.753	841	7	31.1	Similar to H0502B11.4 protein.
Os08g0417100	0.756	224	5	25.5	Similar to cDNA, clone: J100014C05, full insert sequence.
Os05g0133100	0.757	229	2	9.4	Similar to PII protein (Fragment).
Os06g0221300	0.757	227	3	22.8	Similar to Dehydration stress-induced protein.
Os09g0515500	0.758	134	3	5.9	Translation initiation factor 2 related domain containing protein.
Os03g0810800	0.759	360	4	24.2	Similar to Short-chain alcohol dehydrogenase.
Os01g0210500	0.762	1133	6	45	Similar to SOUL-like protein.
Os02g0109100	0.765	668	4	12.1	Similar to diphosphomevalonate decarboxylase.
Os03g0842900	0.768	1047	13	43.9	Similar to Steroleosin-B.
Os03g0111300	0.779	693	3	26	Nonspecific lipid-transfer protein 2 (nsLTP2).
Os04g0614600	0.781	702	7	36.2	Similar to Viroid RNA-binding protein (Fragment).
Os03g0429000	0.782	571	3	37.4	Proteinase inhibitor I25, cystatin domain containing protein.
Os08g0519400	0.782	193	3	10	Apolipoprotein/apolipophorin domain containing protein.
Os05g0301500	0.786	255	3	8.8	Similar to Ribophorin I (Fragment).
Os07g0150200	0.79	1059	1	45.7	Similar to 40S ribosomal protein S12-1.
Os01g0111600	0.793	230	3	20.7	Similar to MOTHER of FT and TF1 protein.
Os05g0557100	0.793	95	4	10.1	Peptidase A1 domain containing protein.
Os04g0107900	0.795	1487	3	26.1	Heat shock protein 81-1 (HSP81-1) (Heat shock protein 83).
Os07g0195400	0.799	496	9	23.7	Phosphoacetylglucosamine mutase domain containing protein.
Os03g0231600	0.799	241	3	9.4	Similar to Branched-chain-amino-acid aminotransferase 3.

Os07g0639900	0.8	205	5	22.5	Thymidylate kinase-like protein.
Os06g0320000	0.8	127	3	30.2	Thioredoxin fold domain containing protein.
Os03g0295800	0.803	634	2	10.9	Gamma interferon inducible GILT protein.
Os04g0165700	0.804	312	3	9.5	Cysteine synthase.
Os05g0457700	0.806	186	4	15.9	Tetratricopeptide-like helical domain containing protein.
Os09g0535300	0.807	124	2	6.6	XAP5 protein family protein.
Os04g0546500	0.807	836	2	13.5	Similar to Oleosin.
Os09g0484200	0.808	2271	7	58	Hypothetical protein.
Os06g0264300	0.809	1082	6	23.7	Similar to RAD23, isoform I.
Os02g0779200	0.81	204	5	10.7	Similar to Subtilisin-like protease (Fragment).
Os06g0112200	0.812	274	3	15.4	Purine and other phosphorylases, family 1 protein.
Os06g0198700	0.813	89	3	18.5	CHD5-like protein domain containing protein.
Os03g0747800	0.815	1563	5	37.8	Cysteine synthase.
Os12g0430000	0.815	414	5	10.2	Hypothetical conserved gene.
Os02g0158900	0.816	244	6	18.8	Similar to SNF4.
Os03g0249300	0.816	137	2	10.8	Nucleotide-sensitive chloride conductance regulator protein.
Os01g0179300	0.817	305	3	8.2	Similar to BRI1-KD interacting protein 128 (Fragment).
Os03g0137500	0.818	227	4	16.3	Similar to VDAC3.1.
Os04g0390800	0.818	2520	16	54.8	NAD(P)-binding domain containing protein.
Os12g0183300	0.819	87	3	23	Similar to 3~(2~),5~-bisphosphate nucleotidase.
Os03g0841700	0.82	622	2	30	Similar to Prohibitin.
Os04g0431100	0.822	112	3	17.6	GrpE protein homolog.
Os08g0549300	0.822	403	1	10.9	Similar to Acyl carrier protein III, chloroplast precursor (ACP III).
Os07g0586200	0.823	201	6	19.4	Similar to Esterase precursor.
Os10g0464400	0.823	142	3	9.3	Haloacid dehalogenase-like hydrolase domain containing protein.
Os04g0165300	0.825	74	2	8.9	Conserved hypothetical protein.

Os05g0346500	0.825	312	6	11.4	Glycoside hydrolase, family 85 domain containing protein.
Os08g0562700	0.826	461	4	11.7	Similar to peptidase M1 family protein.
Os03g0240700	0.826	133	1	10.1	Similar to Erwinia induced protein 2.
Os02g0168800	0.829	143	4	17	Similar to Porphobilinogen deaminase (Fragment).
Os03g0337900	0.831	371	5	13.5	Similar to predicted protein.
Os06g0152100	0.831	1551	2	24.4	Similar to Profilin-2.
Os03g0122200	0.832	78	3	16	Similar to 50S ribosomal protein L11.

Supplementary Table 2. List of proteins up-regulated in embryos of germinated rice seeds in response to BRZ-treatment by iTRAQ

Gene ID	NIP(BRZ / Mock) fold change	Protein score	Unique Peptide	Sequence coverage(%)	Description
Os03g0652100	2.293	233	4	8.2	Not CCR4-Not complex component.
Os01g0815800	2.061	682	1	19.8	Similar to 60S ribosomal protein L24-A.
Os01g0866600	2.018	300	3	54.8	Similar to bolA-like protein.
Os03g0799000	1.997	126	2	18.4	Similar to Histone H1.
Os06g0319700	1.888	170	1	20.2	Similar to 60S ribosomal protein L31.
Os02g0675700	1.741	236	4	18.4	DUF248, methyltransferase putative family protein.
Os05g0486700	1.676	509	1	19.9	Ribosomal protein L24e domain containing protein.
Os12g0498800	1.641	64	2	7.9	Conserved hypothetical protein.
Os03g0708100	1.62	328	1	17.8	Phytanoyl-CoA dioxygenase family protein.
Os06g0705400	1.607	205	1	11.7	Similar to Nonspecific lipid-transfer protein 2P (LTP2P).
Os02g0796900	1.604	98	1	20	Similar to 60S ribosomal protein L39.
Os01g0880800	1.585	891	5	16.8	Similar to Acyl-[acyl-carrier-protein] desaturase.
Os11g0169800	1.576	100	3	11.3	Similar to Long-chain-fatty-acid--CoA ligase 4.

Os03g0579300	1.566	225	1	20.4	60S ribosomal protein L19.
Os07g0119400	1.554	142	4	11.6	Similar to Pectinesterase like protein.
Os05g0555800	1.545	290	2	37.5	Similar to 60S ribosomal protein L35a-3.
Os05g0459900	1.54	407	3	23.9	Similar to 60S ribosomal protein L36-1.
Os10g0437600	1.533	133	3	5.7	Similar to Starch synthase II, chloroplast precursor.
Os07g0691800	1.529	1281	1	37.3	Similar to 26S proteasome subunit 4-like protein.
Os02g0587000	1.529	569	2	20.8	Similar to Glycine rich protein (Fragment).
Os09g0127700	1.506	221	2	21.5	Conserved hypothetical protein.
Os03g0118800	1.486	176	3	12.7	Similar to Hydroxymethylglutaryl-CoA synthase.
Os02g0804100	1.486	242	1	16.1	Similar to predicted protein.
Os07g0184800	1.485	81	1	7.2	Similar to Variant of histone H1.
Os03g0774200	1.484	170	4	18.4	Similar to NADH-ubiquinone oxidoreductase subunit 8.
Os01g0376700	1.472	400	2	11.1	Similar to Sucrose-phosphatase.
Os03g0337800	1.467	356	2	24	Similar to 60S ribosomal protein L19 (Fragment).
Os07g0240300	1.459	96	3	9.9	Similar to OSIGBa0153E02-OSIGBa0093I20.13 protein.
Os09g0402100	1.442	283	4	30.5	PF1 protein.
Os02g0793300	1.423	162	6	9.5	Similar to Nudix hydrolase 3 (AtNUDT3). Splice isoform 2.
Os07g0608700	1.423	124	2	32.5	Similar to small nuclear ribonucleoprotein G.
Os03g0805200	1.421	1077	5	24.6	Similar to RNA helicase (Fragment).
Os02g0329800	1.419	159	3	9.2	Glycosyltransferase AER61.
Os03g0200500	1.411	2811	3	55.3	40S ribosomal protein S3a (CYC07 protein).
Os11g0602200	1.41	219	3	6.4	Similar to SET domain protein SDG111.
Os10g0355800	1.391	2143	4	38.1	Similar to ATP synthase CF1 beta subunit.
Os02g0653800	1.389	996	1	49.8	Similar to GTP-binding protein.
Os12g0506400	1.387	95	1	6.7	Cornichon family protein.
Os06g0150600	1.386	111	3	7.4	Transferase family protein.

Os03g0720300	1.384	452	5	26.6	Similar to Glutamate decarboxylase isozyme 1.
Os12g0166500	1.381	159	4	14.4	Similar to Nrap protein, expressed.
Os02g0550100	1.38	419	1	10.8	Similar to Vacuolar ATP synthase 16 kDa proteolipid subunit.
Os05g0542900	1.378	194	2	7.4	Pectin lyase fold domain containing protein.
Os05g0386800	1.371	66	3	9.2	Similar to Phytochelatin synthetase-like protein.
Os04g0249600	1.371	151	2	23.9	Rhodanese-like domain containing protein.
Os09g0539500	1.367	784	3	36.6	Similar to SKP1-like protein 1A.
Os05g0566500	1.363	372	6	11.8	Similar to Initiation factor 3d (Fragment).
Os03g0368000	1.356	639	5	27.6	Similar to Peroxidase 1.
Os02g0284600	1.353	1167	4	29.2	Similar to 60S ribosomal protein L27.
Os02g0591800	1.347	86	3	13	Brix domain containing protein.
Os01g0360600	1.347	90	5	25.7	Dephospho-CoA kinase family protein.
Os05g0564200	1.338	59	2	8.6	U2 snRNP auxiliary factor, small subunit.
Os05g0105100	1.336	423	1	29.1	Similar to Small GTPase rab11-related.
Os05g0494000	1.334	231	6	13.9	Similar to Cytochrome P450 98A1.
Os11g0168200	1.322	1855	11	28	60S ribosomal protein L3.
Os07g0688800	1.321	248	4	8.5	Aldehyde dehydrogenase domain containing protein.
Os01g0348700	1.319	422	3	20.4	Similar to 60S ribosomal protein L23a (L25).
Os03g0263500	1.318	148	2	9.7	Similar to Splicing factor 3A subunit 2.
Os03g0264400	1.317	186	4	11.2	Anthranilate synthase alpha 2 subunit.
Os08g0101400	1.309	100	3	6.6	Similar to cDNA clone:J023074F23, full insert sequence.
Os03g0823700	1.307	564	3	40.9	Similar to Ras-related protein Rab11C.
Os08g0465800	1.304	952	8	33.4	Similar to Glutamate decarboxylase.
Os08g0555200	1.298	238	3	6.7	Nonaspanin (TM9SF) family protein.
Os05g0103100	1.298	788	3	23.1	Translocon-associated beta family protein.
Os03g0700400	1.297	1056	3	37.3	Similar to LOX4 (Fragment).

Os09g0485900	1.294	1741	1	41.1	Similar to 60S ribosomal protein L9 (Gibberellin-regulated protein).
Os07g0500300	1.293	303	3	32.4	C2 calcium-dependent membrane targeting domain protein.
Os02g0503400	1.29	99	2	13.8	Similar to 60S ribosomal protein L35.
Os03g0750000	1.288	2653	1	25.9	Similar to ethylene-responsive protein.
Os03g0858400	1.285	218	5	14.2	WD40 repeat-like domain containing protein.
Os07g0191200	1.284	1341	4	18.6	Plasma membrane H+ ATPase.
Os08g0117300	1.283	2212	3	57	Similar to 40S ribosomal protein S13.
Os08g0486200	1.283	73	2	5.5	Similar to Splicing factor SC35.
Os03g0154700	1.28	1328	5	27.7	Similar to 40S ribosomal protein S9.
Os04g0497200	1.275	169	4	9.3	Cellulase precursor.
Os08g0178100	1.272	112	4	5.7	Pep3/Vps18/deep orange domain containing protein.
Os11g0171300	1.269	278	4	13.1	Fructose-bisphosphate aldolase, chloroplast precursor.
Os01g0896700	1.268	902	3	34.5	Similar to 60S ribosomal protein L5.
Os01g0140500	1.264	575	2	10.1	Similar to 60S ribosomal protein L26B.
Os02g0199900	1.264	1395	4	43	Similar to 26S proteasome regulatory complex subunit p42D.
Os07g0137900	1.262	176	2	10.3	Hypothetical conserved gene.
Os05g0592400	1.26	485	15	17.2	UV-damaged DNA binding protein.
Os01g0358400	1.259	2745	1	43.4	Similar to 40S ribosomal protein S4.
Os03g0192400	1.258	257	3	26.8	GRIM-19 family protein.
Os02g0586500	1.257	87	1	10.6	Similar to OSIGBa0124N08.5 protein.
Os02g0229000	1.257	774	2	25.4	Similar to 40S ribosomal protein S19-like.
Os07g0490300	1.256	170	2	9.9	Similar to Preproacrosin.
Os10g0571200	1.256	424	6	18.8	Similar to Pyruvate kinase isozyme G, chloroplast (Fragment).
Os07g0448800	1.255	531	5	18.3	Aquaporin.
Os05g0474400	1.253	197	2	10.9	Prenylated rab acceptor PRA1 family protein.
Os03g0379100	1.252	176	3	6.9	DUF248, methyltransferase putative family protein.

Os06g0289900	1.252	1006	9	23.9	UDP-glucuronosyl/UDP-glucosyltransferase family protein.
Os01g0906200	1.251	152	4	13	Similar to heat-intolerant 1.
Os11g0210500	1.25	1940	6	39.8	Similar to Alcohol dehydrogenase.
Os07g0570300	1.248	333	10	16.3	Peptidase M16, core domain containing protein.
Os03g0355600	1.248	333	11	13.7	Similar to Adapter-related protein complex 2 beta 1 subunit.
Os02g0787300	1.248	181	5	18.7	Similar to MAP kinase kinase.
Os03g0226200	1.246	702	3	48.5	Non-symbiotic hemoglobin 2 (rHb2) (ORYsa GLB1b).
Os01g0152300	1.245	737	3	22.9	Similar to Histone H2B.1.
Os03g0267000	1.242	301	2	41	Low molecular mass heat shock protein Oshsp18.0.
Os01g0294700	1.239	707	8	33.7	Haem peroxidase, plant/fungal/bacterial family protein.
Os08g0558600	1.238	105	3	19.1	Synaptobrevin domain containing protein.
Os01g0233000	1.237	391	5	34.3	DREPP plasma membrane polypeptide family protein.
Os05g0103500	1.235	211	1	7.2	CHCH domain containing protein.
Os03g0341100	1.234	506	4	28.3	Similar to 60S ribosomal protein L18.
Os07g0662900	1.233	1172	17	25.3	Similar to 4-alpha-glucanotransferase.
Os09g0487500	1.233	120	1	12.8	Conserved hypothetical protein.
Os08g0562500	1.229	313	6	16.4	Transferase family protein.
Os03g0356484	1.226	150	1	12.1	Tetratricopeptide-like helical domain containing protein.
Os04g0441900	1.225	114	3	6.5	Cleft lip and palate transmembrane 1 family protein.
Os12g0605800	1.225	86	5	8.4	Similar to 3-methylcrotonyl CoA carboxylase.
Os03g0577000	1.222	1613	6	45.6	Similar to Ribosomal protein S3 (Fragment).
Os01g0822900	1.222	1233	3	23.3	Similar to Lipid transfer protein.
Os04g0643100	1.221	192	4	15.6	Similar to Vacuolar ATP synthase subunit D.
Os11g0256050	1.221	177	4	22.1	Hypothetical conserved gene.
Os05g0512600	1.221	365	2	15.4	X8 domain containing protein.
Os02g0797700	1.221	396	5	10.1	Nonaspanin (TM9SF) family protein.

Os07g0180900	1.218	6901	9	40.7	Similar to 60S ribosomal protein L4.
Os02g0105500	1.218	502	4	18.6	IQ calmodulin-binding region domain containing protein.
Os04g0432000	1.217	374	7	26.2	Serine/threonine-protein kinase SAPK7.
Os02g0753300	1.212	1514	4	26.9	Lipoxygenase, LH2 domain containing protein.
Os07g0599000	1.206	218	8	14.3	Pentatricopeptide repeat domain containing protein.
Os05g0302700	1.206	1264	5	21.8	Similar to ATP/ADP carrier protein.
Os09g0298200	1.204	654	4	22.8	Similar to Brittle 2. >Os09t0298200-02 Similar to Brittle 2.
Os06g0103300	1.203	370	3	9.1	Similar to Homogentisate 1,2-dioxygenase.

Supplementary Table 3. List of proteins down-regulated in embryos of germinated rice seeds of mutant *d61-1* by iTRAQ

Gene ID	NIP(BRZ / Mock) fold change	Protein score	Unique Peptide	Sequence coverage(%)	Description
Os04g0308400	0.186	82	1	5.5	Similar to H0211A12.13 protein.
Os03g0266300	0.259	675	2	36.6	Class I low-molecular-weight heat shock protein 17.9.
Os01g0127600	0.276	222	2	15.1	Similar to Bowman-Birk type proteinase inhibitor D-II precursor (IV).
Os02g0268100	0.305	4353	16	60	Similar to Glutelin (Fragment).
Os01g0159600	0.321	2093	3	29	Small hydrophilic plant seed protein family protein.
Os07g0214300	0.321	1815	2	28.3	Seed allergenic protein RAG2 precursor.
Os01g0136000	0.332	197	2	40.3	Similar to Cytosolic class I small heat-shock protein HSP17.5.
Os02g0242600	0.351	1274	7	21.8	Similar to Glutelin.
Os09g0408000	0.356	118	3	7.8	Similar to transducin family protein.
Os05g0268500	0.369	131	5	12	Similar to Serine carboxypeptidase 2.
Os05g0349800	0.375	720	3	24.2	Embryonic abundant protein 1.
Os03g0796000	0.379	218	2	12.6	Similar to Ripening-associated protein (Fragment).

Os08g0320100	0.38	206	4	12.9	Nucleotide-binding, alpha-beta plait domain containing protein.
Os07g0214600	0.383	606	3	25.5	Similar to Seed allergenic protein RA17 precursor.
Os04g0395700	0.389	121	1	7.5	Conserved hypothetical protein.
Os07g0213800	0.392	507	5	37.5	Similar to Allergenic protein.
Os09g0127700	0.4	221	2	21.5	Conserved hypothetical protein.
Os02g0249000	0.411	804	10	22.7	Glutelin, Seed strage protein
Os02g0453600	0.414	539	5	28.1	Similar to Glutelin.
Os05g0329100	0.421	917	3	42.7	Prolamin.
Os02g0175800	0.428	93	4	34.7	Similar to fiber protein Fb15.
Os03g0804200	0.429	186	3	22.1	Bifunctional inhibitor/plant lipid transfer protein.
Os01g0828100	0.44	130	2	9.5	Similar to Cinnamoyl-CoA reductase.
Os01g0975900	0.445	74	1	6.3	Similar to Tonoplast membrane integral protein ZmTIP1-2.
Os02g0249800	0.45	3900	2	36.3	Glutelin precursor.
Os06g0625400	0.466	86	3	5.1	Similar to Metalloendopeptidase.
Os04g0434400	0.469	348	4	10.3	Similar to H0823A09.7 protein.
Os09g0109600	0.471	167	2	40	Conserved hypothetical protein.
Os10g0349400	0.473	104	1	9.5	Similar to cortical cell-delineating protein.
Os09g0324000	0.473	181	2	22.2	Similar to Oleosin.
Os01g0136100	0.479	1226	2	40	16.9 kDa class I heat shock protein 1.
Os05g0113400	0.481	150	2	20.3	Similar to Actin-depolymerizing factor 2 (ADF 2).
Os05g0477900	0.494	811	1	16.9	Similar to nonspecific lipid-transfer protein.
Os04g0593400	0.506	94	1	9.3	Similar to OSIGBa0142I02-OSIGBa0101B20.20 protein.
Os01g0225600	0.506	414	3	30.5	Similar to Dehydrin.
Os01g0705200	0.521	2326	8	36.7	Late embryogenesis abundant protein.
Os03g0159600	0.529	1489	6	49.6	Similar to Rab28 protein.
Os05g0595400	0.53	545	5	21.3	Similar to Nucleoside diphosphate kinase III

Os06g0114400	0.532	235	1	7.5	Conserved hypothetical protein.
Os08g0473600	0.536	289	3	10.1	Alpha-amylase isozyme 3E precursor.
Os02g0611800	0.541	252	2	10	Similar to Hydroxyanthranilate hydroxycinnamoyltransferase 3.
Os02g0123500	0.545	191	6	21.9	Similar to NADPH-dependent mannose 6-phosphate reductase.
Os07g0422100	0.551	260	1	9.2	AWPM-19-like family protein.
Os04g0322100	0.553	308	4	21	Protein of unknown function DUF26 domain containing protein.
Os03g0306900	0.559	121	2	9	Haem oxygenase-like, multi-helical domain containing protein.
Os10g0361900	0.56	1926	2	31.1	Lipoxygenase, LH2 domain containing protein.
Os02g0765600	0.561	660	9	32.5	Alpha-amylase glycoprotein, Degradation of starch granule
Os03g0734200	0.566	254	1	17.4	Conserved hypothetical protein.
Os02g0782500	0.567	149	3	15.1	Similar to Small heat stress protein class CIII.
Os10g0437500	0.567	1548	5	38.1	Rossmann-like alpha/beta sandwich fold domain containing protein.
Os02g0249600	0.568	3780	1	34.9	Similar to Glutelin.
Os05g0341600	0.57	57	2	17.9	Similar to Ras-related protein Rab-21.
Os06g0133000	0.57	495	9	22.2	Granule-bound starch synthase I, chloroplast precursor.
Os07g0215500	0.57	616	2	25	Allergenic protein.
Os05g0133100	0.571	229	2	9.4	Similar to PII protein (Fragment).
Os01g0228600	0.572	139	2	9.8	Similar to 2-hydroxyacid dehydrogenase (AGR_L_379p).
Os02g0567800	0.573	72	4	13.8	Alpha/beta hydrolase fold-3 domain containing protein.
Os07g0557700	0.574	76	2	8.7	Conserved hypothetical protein.
Os07g0694500	0.574	181	4	8.5	Similar to PWWP domain containing protein.
Os04g0404400	0.577	841	7	31.1	Similar to H0502B11.4 protein.
Os01g0666800	0.583	223	4	5.8	Similar to predicted protein.
Os10g0416800	0.583	120	4	20.8	Similar to Chitinase 2 (EC 3.2.1.14) (Tulip bulb chitinase-2) (TBC-2).
Os04g0445100	0.586	460	4	25.1	Similar to 22.7 kDa class IV heat shock protein precursor.
Os11g0620300	0.589	254	3	30.9	Similar to Nonspecific lipid-transfer protein 2 (LTP 2).

Os07g0237100	0.59	121	2	8.5	RNA recognition motif domain domain containing protein.
Os03g0842900	0.592	1047	13	43.9	Similar to Steroleosin-B.
Os10g0177200	0.594	221	5	14.5	EF-HAND 2 domain containing protein.
Os02g0589400	0.599	78	3	11.1	UDP-glucuronosyl/UDP-glucosyltransferase family protein.
Os11g0213600	0.6	180	5	11.6	Peptidase S10, serine carboxypeptidase family protein.
Os06g0704600	0.601	284	7	19.7	Similar to Delta-aminolevulinic acid dehydratase (Fragment).
Os07g0644000	0.603	128	3	12.8	ATP12, ATPase F1F0-assembly protein domain containing protein.
Os03g0219300	0.603	1691	2	35	Similar to Tubulin alpha-2 chain (Alpha-2 tubulin).
Os04g0473150	0.604	703	2	22.2	Similar to photosystem II protein D1.
Os05g0453700	0.607	353	6	59.4	Similar to ENOD18 protein (Fragment).
Os04g0486950	0.608	721	11	26.3	Similar to Malate synthase.
Os01g0119100	0.608	58	3	5.9	Similar to Glycosyltransferase.
Os06g0705400	0.609	205	1	11.7	Similar to Nonspecific lipid-transfer protein 2P (LTP2P).
Os03g0267000	0.613	301	2	41	Low molecular mass heat shock protein Oshsp18.0.
Os04g0676100	0.613	79	2	13.9	Similar to Thioredoxin X, chloroplast precursor.
Os05g0468800	0.615	616	4	31.1	Phosphatidylethanolamine-binding protein PEBP domain protein.
Os03g0689300	0.615	1666	4	21.4	Plasma membrane H+ ATPase (EC 3.6.3.6) (H-ATPase).
Os10g0491000	0.616	124	2	9.2	Plant Basic Secretory Protein family protein.
Os02g0158900	0.626	244	6	18.8	Similar to SNF4.
Os01g0907600	0.628	290	4	13.7	Cysteine endopeptidase.
Os04g0118400	0.628	7065	1	51.2	Similar to Elongation factor EF-2 (Fragment).
Os01g0210500	0.633	1133	6	45	Similar to SOUL-like protein.
Os04g0629500	0.635	143	1	6.8	Similar to Thioredoxin h.
Os01g0598600	0.636	654	9	32.6	Peptidase A1 domain containing protein.
Os03g0605300	0.639	135	3	5.2	Similar to Subtilisin-like protease (Fragment).
Os12g0125400	0.644	667	2	25.3	Similar to Homoserine dehydrogenase-like protein.

Os07g0485100	0.652	195	3	11.4	Similar to Beta-ureidopropionase (Beta-alanine synthase).
Os01g0111600	0.653	230	3	20.7	Similar to MOTHER of FT and TF1 protein.
Os03g0663400	0.654	64	2	10.8	Similar to Thaumatin-like protein.
Os03g0130300	0.656	122	2	27.2	Similar to Cp-thionin.
Os10g0191300	0.66	151	2	18.2	Similar to PR-1a pathogenesis related protein (Hv-1a) precursor.
Os12g0430000	0.662	414	5	10.2	Hypothetical conserved gene.
Os03g0134900	0.664	119	5	17.6	Similar to Glutathione S-transferase GSTF14 (Fragment).
Os03g0381500	0.667	637	2	17.1	Conserved hypothetical protein.
Os06g0246500	0.668	494	8	30.4	Similar to Pyruvate dehydrogenase E1 alpha subunit.
Os12g0178200	0.668	266	2	24.1	Similar to Thylakoid-bound ascorbate peroxidase (Fragment).
Os04g0513100	0.668	273	2	6.2	Similar to Beta-glucosidase.
Os01g0613300	0.668	97	3	19.2	Conserved hypothetical protein CHP02058 domain protein.
Os04g0390800	0.669	2520	16	54.8	NAD(P)-binding domain containing protein.
Os04g0661600	0.675	357	6	22.7	Similar to H0112G12.13 protein.
Os04g0674700	0.675	184	3	7.3	Similar to AMP-binding protein.
Os06g0134800	0.676	635	6	20.7	Folate-binding, YgfZ domain containing protein.
Os06g0726400	0.678	591	12	15.7	Branching enzyme-I precursor (Starch-branching enzyme I).
Os12g0626500	0.679	1531	3	26.8	Similar to late embryogenesis abundant protein D-34.
Os01g0651800	0.679	209	3	10	Lipase, class 3 family protein.
Os03g0804700	0.679	158	2	12.4	Germin-like protein 3-8.
Os03g0411300	0.679	427	1	7	EF-Hand type domain containing protein.
Os12g0555500	0.682	986	9	60.1	Probenazole-inducible protein PBZ1.
Os04g0685200	0.682	753	7	32.1	Peptidase aspartic, catalytic domain containing protein.
Os02g0595500	0.683	862	5	34.4	Similar to NAD-dependent isocitrate dehydrogenase precursor.
Os04g0526600	0.684	1430	7	41.5	Similar to Alpha-amylase/subtilisin inhibitor (RASI).
Os07g0468100	0.684	655	7	34.5	Similar to Glutathione S-transferase GST 19.

Os09g0535000	0.686	1153	10	46.1	Similar to Triosephosphate isomerase, chloroplast precursor.
Os08g0549300	0.687	403	1	10.9	Similar to Acyl carrier protein III, chloroplast precursor (ACP III).
Os04g0665800	0.689	370	3	14	Similar to H1005F08.12 protein.
Os04g0127300	0.691	99	6	14.9	Peptidase S8 and S53, subtilisin, kexin, sedolisin domain protein.
Os02g0204000	0.692	92	2	10.7	Tetratricopeptide-like helical domain containing protein.
Os11g0149200	0.692	201	1	7.5	Conserved hypothetical protein.
Os06g0625500	0.693	3443	7	43.5	Similar to Thioredoxin peroxidase.
Os08g0345800	0.695	289	5	22.5	Similar to Glucose-1-phosphate adenylyltransferase small subunit.
Os03g0113700	0.696	2358	3	24.9	Similar to Heat shock 70 kDa protein, mitochondrial precursor.
Os01g0921600	0.697	648	6	40.1	Similar to Mitochondrial import receptor subunit TOM20.
Os11g0568500	0.698	211	3	36.1	Prefoldin domain containing protein.
Os09g0514600	0.7	521	4	23.4	Beta-grasp fold, ferredoxin-type domain containing protein.
Os04g0679400	0.701	341	7	41.9	Similar to H0801D08.15 protein.
Os03g0659300	0.702	1909	5	46.1	Glyoxalase/bleomycin resistance protein/dioxygenase.
Os03g0790900	0.703	1130	18	20.4	Similar to Aldehyde oxidase-2.
Os03g0141200	0.704	147	6	16	Similar to Beta-amylase PCT-BMYI.
Os05g0457700	0.705	186	4	15.9	Tetratricopeptide-like helical domain containing protein.
Os09g0568900	0.706	271	1	23.2	Similar to predicted protein.
Os11g0701100	0.708	174	4	21	Similar to Class III chitinase homologue (OsChib3H-h) (Fragment).
Os06g0714100	0.71	346	5	53.1	Complex 1 LYR protein family protein.
Os10g0406600	0.712	513	4	47.8	Complex 1 LYR protein family protein.
Os07g0159800	0.714	296	1	8.6	MD-2-related lipid-recognition domain containing protein.
Os05g0358400	0.715	115	3	11.4	Butirosin biosynthesis, BtrG-like domain containing protein.
Os09g0539500	0.716	784	3	36.6	Similar to SKP1-like protein 1A.
Os06g0105400	0.717	309	3	17.5	Similar to dihydrolipoamide S-acetyltransferase.
Os04g0543900	0.719	404	5	21.4	Similar to Glutamate dehydrogenase 2.

Os06g0498400	0.723	884	21	18.5	Similar to Alpha-glucan water dikinase, chloroplast precursor.
Os06g0325500	0.725	233	4	10.4	Uncharacterised conserved protein UCP030210.
Os09g0441400	0.725	97	4	8.4	Similar to Elicitor-inducible cytochrome P450.
Os11g0250000	0.726	268	3	20.2	Similar to RNA recognition motif family protein, expressed.
Os07g0639800	0.728	392	5	31.8	Similar to Eukaryotic translation initiation factor 6 (Fragment).
Os08g0338700	0.728	711	4	24.1	Similar to Chaperone GrpE type 2.
Os02g0664200	0.732	76	3	9.9	Similar to UDP-glucose:glycoprotein glucosyltransferase 1 precursor.
Os05g0557200	0.733	758	9	23.2	Armadillo-type fold domain containing protein.
Os11g0115400	0.734	379	2	25.9	Lipid transfer protein LPT IV.
Os03g0210600	0.734	266	5	24.3	Conserved hypothetical protein.
Os04g0497000	0.734	52	3	11.3	Similar to Allyl alcohol dehydrogenase.
Os09g0382500	0.737	112	2	12.2	Conserved hypothetical protein.
Os03g0295500	0.737	93	1	12.7	CHCH domain containing protein.
Os05g0399300	0.738	290	3	24.2	Similar to Chitinase.
Os03g0197400	0.739	475	5	17.8	Similar to COP9 signalosome complex subunit 4.
Os03g0101600	0.74	879	10	37.7	Similar to Oxidoreductase, zinc-binding dehydrogenase protein.
Os09g0516200	0.742	418	3	7.1	Similar to Transcription factor RF2a.
Os03g0405100	0.743	467	6	22.9	Ubiquinone biosynthesis protein COQ9 domain containing protein.
Os10g0320400	0.743	942	8	28.1	Similar to ATP synthase gamma chain, mitochondrial precursor.
Os05g0460000	0.743	12574	2	46.3	Similar to 70 kDa heat shock cognate protein 1.
Os03g0735300	0.745	496	6	23.3	Alba, DNA/RNA-binding protein family protein.
Os07g0104500	0.746	383	5	16.1	Haem peroxidase, plant/fungal/bacterial family protein.
Os03g0277500	0.747	2863	6	56.2	Similar to Glyoxalase family protein, expressed.
Os07g0213600	0.747	238	2	24.7	Bifunctional inhibitor/plant lipid transfer protein.
Os03g0655700	0.747	516	8	33.3	Similar to 3-isopropylmalate dehydrogenase 2.
Os01g0918200	0.748	297	1	20.8	Similar to Ubiquitin-like protein SMT3.

Os03g0820500	0.749	1206	3	34.7	Similar to WCOR719.
Os02g0595700	0.75	719	9	25.5	Chloroplast translational elongation factor Tu.
Os03g0426900	0.751	1281	16	25.3	Similar to Heat shock protein 101.
Os01g0811100	0.753	133	4	21.3	Proteasome subunit alpha type 3.
Os10g0155500	0.754	357	8	27.9	Similar to Aldose 1-epimerase-like protein.
Os05g0561500	0.754	294	4	24.9	Similar to CMP-KDO synthetase (Fragment).
Os08g0430700	0.754	1046	5	20.8	Similar to UVB-resistance protein-like.
Os01g0351300	0.756	175	8	13.5	Exocyst complex subunit Sec15-like family protein.
Os06g0215600	0.756	271	3	13.1	Similar to Oxo-phytodienoic acid reductase.
Os10g0411700	0.758	464	1	16.9	Similar to S28 ribosomal protein (Fragment).
Os06g0584200	0.758	183	4	7.7	N-6 adenine-specific DNA methylase, conserved site domain protein.
Os09g0133200	0.759	154	5	23.3	Similar to Dehydrogenase/reductase SDR family member 4.
Os04g0117900	0.76	1097	5	27.6	Amidase family protein.
Os08g0250200	0.761	287	2	42	ATPase, F1 complex, epsilon subunit, mitochondrial family protein.
Os01g0531500	0.761	2211	6	33.1	Dienelactone hydrolase domain containing protein.
Os02g0282100	0.761	65	2	17.4	Conserved hypothetical protein.
Os04g0479200	0.762	254	3	23.9	Similar to NAD-dependent isocitrate dehydrogenase c;1.
Os02g0715400	0.767	349	2	32.7	Conserved hypothetical protein.
Os06g0114500	0.769	489	1	16.5	Similar to ATOZI1 protein (Stress-induced protein OZI1).
Os02g0276400	0.77	117	2	11.9	Similar to Isochorismatase family protein rutB.
Os09g0315700	0.771	1265	11	27.5	Phosphoenolpyruvate carboxylase family protein.
Os04g0338000	0.771	457	12	35.3	Similar to IN2-2 protein.
Os07g0195400	0.771	496	9	23.7	Phosphoacetylglucosamine mutase domain containing protein.
Os09g0548200	0.772	672	2	8.6	Peptidoglycan-binding Lysin subgroup domain containing protein.
Os04g0390700	0.773	1162	11	38.3	Short-chain dehydrogenase/reductase SDR domain protein.
Os05g0100800	0.773	215	1	8.2	Nucleic acid-binding, OB-fold domain containing protein.

Os01g0763300	0.773	189	5	58.4	Conserved hypothetical protein.
Os07g0640100	0.773	478	3	20.6	Complex 1 LYR protein family protein.
Os03g0337800	0.775	356	2	24	Similar to 60S ribosomal protein L19 (Fragment).
Os01g0626300	0.775	212	3	61.2	Similar to Mitochondrial import receptor subunit TOM7-1.
Os03g0761000	0.777	619	3	22.9	SWIB/MDM2 domain containing protein.
Os01g0753100	0.777	614	8	31.5	Alcohol dehydrogenase superfamily, zinc-containing protein.
Os03g0707900	0.778	60	3	6.3	Similar to Toc64.
Os12g0620400	0.778	644	4	24.4	Methyl-CpG DNA binding domain containing protein.
Os02g0741500	0.778	243	5	14.2	Ribbon-helix-helix domain containing protein.
Os09g0460400	0.778	140	4	18.4	Alpha/beta hydrolase fold-3 domain containing protein.
Os03g0839100	0.779	181	6	13.7	Ubiquinone biosynthesis hydroxylase.
Os03g0295800	0.78	634	2	10.9	Gamma interferon inducible lysosomal thiol reductase GILT protein.
Os03g0385400	0.781	882	4	30.3	Bifunctional inhibitor/plant lipid transfer protein.
Os06g0675700	0.781	831	4	18	Similar to High pI alpha-glucosidase.
Os01g0939700	0.783	468	7	28.1	Similar to Esterase D.
Os04g0480100	0.783	486	8	22.4	Eukaryotic translation initiation factor 4B.
Os05g0405000	0.783	1494	14	22.5	Orthophosphate dikinase precursor.
Os01g0558300	0.784	168	3	13.4	RWD domain containing protein.
Os04g0310500	0.784	445	4	47.5	NADH dehydrogenase (complex I).
Os02g0173900	0.785	937	4	20.6	Similar to SSADH.
Os03g0218500	0.786	614	9	24.9	Similar to 70kD heat shock protein.
Os04g0173800	0.786	2913	4	33.9	Lectin precursor (Agglutinin).
Os05g0209600	0.786	278	6	20.8	Similar to Esterase.
Os07g0205000	0.786	341	4	28	Similar to Cytochrome-C reductase 14 kDa subunit (Fragment).
Os05g0508500	0.787	857	3	35.5	Similar to Thioredoxin H.
Os03g0212700	0.787	1795	11	28.7	Similar to Cytochrome C reductase-processing peptidase subunit I.

Os09g0474300	0.787	466	4	24.9	Similar to Heat-shock protein precursor.
Os06g0103300	0.788	370	3	9.1	Similar to Homogentisate 1,2-dioxygenase.
Os04g0527900	0.789	384	3	12.1	Similar to Tonoplast membrane integral protein ZmTIP3-2.
Os03g0332400	0.79	362	4	17.4	Similar to Hydroxyacylglutathione hydrolase cytoplasmic (Glx II).
Os12g0512800	0.791	100	5	10.5	Cytochrome P450 71E1.
Os12g0230100	0.793	860	10	25.4	Similar to ATP-dependent Clp protease ATP-binding subunit clpA.
Os10g0492101	0.794	243	2	17.7	Hypothetical conserved gene.
Os02g0611400	0.794	566	8	16.5	Pentatricopeptide repeat domain containing protein.
Os04g0482000	0.794	123	3	17.1	Similar to Peptide methionine sulfoxide reductase.
Os03g0233900	0.795	345	3	41.6	Non-symbiotic hemoglobin 1 (rHb1) (ORYsa GLB1a).
Os12g0616200	0.797	233	3	17.1	HUELLENLOS-like protein.
Os08g0151800	0.797	601	7	19.1	FAD-dependent pyridine nucleotide-disulphide oxidoreductase.
Os02g0706500	0.797	175	3	9.4	ATPase, AAA-type, core domain containing protein.
Os01g0266600	0.798	320	4	33.3	Thioredoxin fold domain containing protein.
Os03g0758400	0.799	234	3	15.1	Ankyrin repeat containing protein.
Os06g0320000	0.8	127	3	30.2	Thioredoxin fold domain containing protein.
Os10g0447100	0.8	60	1	6.9	Similar to Ubiquitin-conjugating enzyme E2-21 kDa 2.
Os01g0184100	0.801	1411	4	30.7	Similar to 17.5 kDa class II heat shock protein.
Os04g0106300	0.801	534	7	29.4	Similar to Arginase.
Os10g0415600	0.804	510	8	12.1	Similar to Prolyl oligopeptidase family protein.
Os03g0313000	0.804	450	5	31.2	Similar to NADH-ubiquinone oxidoreductase (Fragment).
Os01g0168100	0.804	134	5	15.3	KIP1-like domain containing protein.
Os07g0617800	0.804	1298	10	39.2	Similar to Alanine aminotransferase.
Os05g0519700	0.805	8328	26	43.6	Heat shock protein 101.
Os03g0854400	0.805	987	4	32	Ribonuclease III domain containing protein.
Os10g0411800	0.806	4046	5	56	Similar to 40S ribosomal protein S17-3.

Os05g0303000	0.807	642	8	25.5	Similar to Chloroplast heat shock protein 70.
Os03g0390400	0.807	539	4	39.1	Similar to Cytochrome c oxidase subunit 6b.
Os02g0162500	0.808	2708	5	38.7	Similar to 40S ribosomal protein S14.
Os05g0584200	0.808	375	3	26.5	Similar to Late embryogenesis abundant protein Lea14-A.
Os03g0712700	0.809	4568	21	48.5	Similar to Phosphoglucomutase, cytoplasmic 2.
Os08g0536000	0.809	711	6	25.1	Similar to Pyruvate dehydrogenase E1 beta subunit isoform 1.
Os06g0214850	0.81	437	12	47.7	Similar to gibberellin receptor GID1L2.
Os05g0155300	0.81	305	4	18.4	Similar to HIRA interacting protein 5.
Os02g0188000	0.813	545	3	10.5	UDP-glucuronosyl/UDP-glucosyltransferase family protein.
Os02g0143100	0.813	769	7	23.9	Similar to Sucrose-phosphatase.
Os07g0639000	0.813	2153	8	48	Similar to Class III peroxidase 46.
Os01g0665400	0.814	185	3	10.6	Ribokinase family protein.
Os09g0338400	0.815	515	8	26.2	Similar to Cysteine desulfurase, mitochondrial precursor.
Os06g0531200	0.815	1511	13	45.4	ThiJ/PfpI domain containing protein.
Os02g0205200	0.815	495	8	28.8	Similar to Unidentified precursor.
Os09g0123200	0.815	214	4	8.7	Similar to Flowering time control protein isoform OsFCA-1.
Os02g0530600	0.815	3497	26	41.3	Poly(ADP-ribose) polymerase, regulatory region domain protein.
Os03g0399800	0.816	576	5	41.2	Mannose-binding lectin domain containing protein.
Os07g0570700	0.816	109	2	12.4	Ribosome recycling factor family protein.
Os01g0618100	0.817	374	6	24.7	Similar to IN2-2 protein.
Os01g0217800	0.818	323	5	17.1	ThiJ/PfpI domain containing protein.
Os07g0640200	0.82	324	8	19	Carbohydrate kinase, FGGY family protein.
Os10g0417600	0.821	1190	8	51.1	NAD(P)-binding domain containing protein.
Os05g0140500	0.821	260	2	17.2	RNA binding motif protein 8 family protein.
Os02g0257200	0.821	497	4	10.5	Conserved hypothetical protein.
Os05g0547850	0.823	289	2	16.4	DNA-binding TFAR19-related protein family protein.

Os03g0780500	0.823	665	3	24.6	Similar to inosine-5-monophosphate dehydrogenase 2.
Os03g0776000	0.823	2107	2	33.7	Glucose-6-phosphate isomerase, cytosolic A (GPI-A).
Os02g0187100	0.823	770	4	21.8	Similar to cyclase.
Os02g0753300	0.826	1514	4	26.9	Lipoxygenase, LH2 domain containing protein.
Os12g0632700	0.826	1165	6	20.5	Malate dehydrogenase, glyoxysomal precursor.
Os04g0650000	0.827	771	4	23.5	Similar to cDNA clone:J013002H09, full insert sequence.
Os06g0668200	0.827	8132	11	64.1	Similar to Phosphoglycerate kinase, cytosolic.
Os04g0439900	0.828	940	10	31.2	Similar to Translocon Tic40 precursor.
Os08g0440800	0.832	1186	10	29.7	Glyceraldehyde-3-phosphate dehydrogenase.
Os01g0849600	0.832	593	7	62.3	Similar to ENOD18 protein (Fragment).
Os06g0258900	0.832	766	18	16.9	NAD(P)-binding domain containing protein.

Supplementary Table 4. List of proteins up-regulated in embryos of germinated rice seeds of mutant *d61-1* by iTRAQ

Gene ID	NIP(BRZ / Mock) fold change	Protein score	Unique Peptide	Sequence coverage(%)	Description
Os01g0124650	4.341	99	1	7.6	Hypothetical conserved gene.
Os07g0124900	2.796	93	1	7.8	Allergen V5/Tpx-1 related family protein.
Os01g0695100	2.618	144	2	49	Similar to Phosphoethanolamine N-methyltransferase.
Os10g0528300	2.393	136	5	23.4	Tau class GST protein 4.
Os03g0802500	2.316	161	4	8.1	ATPase, AAA-type, core domain containing protein.
Os09g0491772	2.314	1982	11	28.8	Similar to Heat shock protein 70 (Hsc70-5).
Os01g0815800	2.224	682	1	19.8	Similar to 60S ribosomal protein L24-A (L30A) (RP29) (YL21).
Os05g0548900	2.215	200	2	8.7	Similar to Phosphoethanolamine methyltransferase.

Os02g0704900	2.176	385	2	31.3	Similar to Inorganic pyrophosphatase-like protein.
Os02g0804100	2.092	242	1	16.1	Similar to predicted protein.
Os05g0552300	2.09	777	6	32.7	Similar to Guanine nucleotide-binding protein (GPB-LR) (RWD).
Os04g0623500	2.031	201	3	11	Similar to H0215F08.7 protein.
Os03g0799000	2.018	126	2	18.4	Similar to Histone H1.
Os03g0835800	2.004	153	3	11.3	Hypothetical conserved gene.
Os12g0189300	1.93	625	8	28.4	Pyruvate/Phosphoenolpyruvate kinase.
Os10g0361000	1.89	3750	1	28.7	Lipoxygenase, LH2 domain containing protein.
Os03g0720300	1.884	452	5	26.6	Similar to Glutamate decarboxylase isozyme 1.
Os01g0880800	1.835	891	5	16.8	Similar to Acyl-[acyl-carrier-protein] desaturase.
Os03g0350300	1.806	1061	2	36.6	Similar to SAR DNA-binding protein-like protein.
Os03g0118800	1.8	176	3	12.7	Similar to Hydroxymethylglutaryl-CoA synthase.
Os02g0529700	1.792	550	3	61.1	Similar to Acidic ribosomal protein P2a-4 (Fragment).
Os07g0207400	1.79	1876	3	50.3	Similar to RF12 protein (Fragment).
Os02g0714200	1.781	464	5	23.2	Similar to Pyrophosphate--fructose 6-phosphate 1-phosphotransferase alpha subunit .
Os05g0135700	1.769	1911	4	52.3	S-adenosylmethionine synthetase 1.
Os06g0194900	1.756	6017	21	44.3	Sucrose synthase 2.
Os11g0602200	1.744	219	3	6.4	Similar to SET domain protein SDG111.
Os03g0796501	1.728	2480	5	34.2	Similar to 60S ribosomal protein L4 (L1).
Os02g0550100	1.726	419	1	10.8	Similar to Vacuolar ATP synthase 16 kDa proteolipid subunit.
Os08g0488100	1.725	117	1	17.2	Similar to Cortical cell-delineating protein.
Os01g0896700	1.701	902	3	34.5	Similar to 60S ribosomal protein L5.
Os02g0735200	1.686	424	5	36	Glutamine synthetase shoot isozyme.
Os06g0611900	1.682	150	6	13.6	Similar to Glycine decarboxylase P subunit.
Os10g0571200	1.679	424	6	18.8	Similar to Pyruvate kinase isozyme G, chloroplast (Fragment).

Os06g0168600	1.664	273	6	13	Ribonucleotide reductase, Chloroplast biogenesis
Os10g0112600	1.662	326	2	8	Nonaspanin (TM9SF) family protein.
Os07g0545400	1.654	174	1	23.7	Protein H2A.
Os12g0625000	1.647	352	3	19.6	Similar to Cysteine synthase.
Os06g0614300	1.631	163	3	9	C-CAP/cofactor C-like domain domain containing protein.
Os10g0397400	1.622	147	4	19.1	Similar to Cell elongation protein DIMINUTO.
Os03g0736300	1.621	99	4	6.5	Similar to CEL6=CELLULASE 6 (Fragment).
Os02g0675700	1.617	236	4	18.4	DUF248, methyltransferase putative family protein.
Os09g0491100	1.604	162	7	17	Similar to Beta-primeverosidase.
Os01g0150000	1.603	136	4	11.9	3-oxo-5-alpha-steroid 4-dehydrogenase.
Os03g0352300	1.603	1016	10	24.3	Similar to Nucleolar protein.
Os03g0237000	1.587	528	6	15.3	Nonaspanin (TM9SF) family protein.
Os03g0401300	1.584	7456	15	42.3	Sucrose synthase 2.
Os11g0210500	1.581	1940	6	39.8	Similar to Alcohol dehydrogenase.
Os01g0375000	1.578	78	6	12.4	GTP1/OBG domain containing protein.
Os08g0127900	1.576	680	6	12.8	Similar to Globulin 1 (Fragment).
Os04g0474800	1.565	442	6	28.1	Similar to Isoform 2 of Beta-glucosidase 12.
Os08g0104400	1.564	276	2	31.2	Conserved hypothetical protein.
Os07g0500300	1.563	303	3	32.4	C2 calcium-dependent membrane targeting domain protein.
Os02g0718900	1.557	1290	5	26.7	ADP,ATP carrier protein, mitochondrial precursor.
Os06g0604200	1.556	732	5	18.3	Phospholipase D.
Os04g0612600	1.555	107	2	11.5	Similar to Coatomer-like protein, epsilon subunit.
Os01g0348700	1.552	422	3	20.4	Similar to 60S ribosomal protein L23a (L25).
Os05g0388600	1.55	157	5	16.7	Protein of unknown function DUF3411 domain containing protein.
Os05g0503300	1.55	239	9	16.8	Similar to Sulfite reductase (Fragment).
Os01g0860300	1.546	655	2	28.2	Similar to Ribosomal protein L1.

Os12g0443500	1.54	4492	2	53.8	Similar to UDP-glucose 6-dehydrogenase.
Os03g0700700	1.539	1028	18	24.9	Similar to Lipoxygenase (Fragment).
Os02g0788800	1.528	112	2	8.2	Similar to amino acid transporter family protein.
Os02g0126700	1.528	190	3	36.4	Similar to Small nuclear ribonucleoprotein homolog.
Os01g0205500	1.523	1589	1	33.5	Similar to 60S ribosomal protein L11-2 (L16). Splice isoform 2.
Os01g0166700	1.518	87	1	5.3	Saposin family protein.
Os03g0280800	1.509	140	1	7.9	Similar to UDP-D-glucuronate decarboxylase.
Os11g0168200	1.503	1855	11	28	60S ribosomal protein L3.
Os09g0482100	1.502	5736	2	41.3	Similar to Heat shock protein 82.
Os07g0638400	1.5	142	5	34.5	Similar to 1-Cys peroxiredoxin.
Os02g0285300	1.499	407	3	19	DREPP plasma membrane polypeptide family protein.
Os11g0704600	1.498	248	3	9.1	Similar to Beta-1,3 glucanase precursor.
Os09g0482400	1.495	6307	2	41.3	Similar to Heat shock protein 81-3.
Os05g0362500	1.494	149	1	11	Protein of unknown function DUF538 domain containing protein.
Os05g0494000	1.492	231	6	13.9	Similar to Cytochrome P450 98A1.
Os02g0146600	1.487	3896	3	50	Similar to Eukaryotic initiation factor 4A (eIF4A) (eIF-4A).
Os02g0192500	1.485	109	5	10.6	Similar to Cellulose synthase-like protein (Fragment).
Os03g0130400	1.482	193	4	20.8	Adenylate kinase, subfamily protein.
Os10g0486900	1.477	71	2	9.3	Tetratricopeptide-like helical domain containing protein.
Os01g0358400	1.475	2745	1	43.4	Similar to 40S ribosomal protein S4.
Os05g0555800	1.474	290	2	37.5	Similar to 60S ribosomal protein L35a-3.
Os07g0608700	1.474	124	2	32.5	Similar to small nuclear ribonucleoprotein G.
Os02g0209000	1.473	138	6	10.2	Hypothetical conserved gene.
Os05g0563550	1.472	382	3	14.3	FAS1 domain domain containing protein.
Os05g0597100	1.469	301	4	15.5	Similar to Nucleolar histone deacetylase HD2-p39.
Os04g0165700	1.467	312	3	9.5	Cysteine synthase.

Os02g0822200	1.46	135	2	5.7	Peptidase C12, ubiquitin carboxyl-terminal hydrolase 1 protein.
Os02g0713400	1.458	503	5	29.9	Similar to Thioredoxin reductase 1 (NTR 1).
Os05g0486700	1.457	509	1	19.9	Ribosomal protein L24e domain containing protein.
Os11g0169800	1.457	100	3	11.3	Similar to Long-chain-fatty-acid--CoA ligase 4 (LACS 4).
Os02g0591800	1.451	86	3	13	Brix domain containing protein.
Os07g0586500	1.449	141	5	14.3	Similar to SUMO activating enzyme 2.
Os01g0188400	1.449	1110	4	15.5	NADP-dependent malic enzyme, chloroplast precursor (NADP-ME).
Os06g0731800	1.448	309	1	5.2	Clathrin light chain family protein.
Os12g0548401	1.447	999	4	54.5	Similar to Proteinase inhibitor.
Os04g0533300	1.445	153	3	19.4	Similar to Remorin (pp34).
Os07g0182100	1.444	272	7	30.1	Similar to Tryptophan synthase alpha chain.
Os11g0216400	1.44	303	2	8.6	Peptidase S8, subtilisin-related domain containing protein.
Os10g0555900	1.433	344	5	28.7	Beta-expansin precursor.
Os11g0703900	1.432	16099	4	48.8	Heat shock protein 70.
Os04g0462500	1.43	4933	1	54.2	Similar to 14-3-3-like protein GF14-6.
Os03g0288600	1.427	107	3	8	Muscle derived-like protein.
Os02g0717400	1.425	875	15	15.7	Tetratricopeptide-like helical domain containing protein.
Os03g0823700	1.421	564	3	40.9	Similar to Ras-related protein Rab11C.
Os06g0656500	1.42	160	3	13.8	Similar to 4-coumarate--CoA ligase 1.
Os07g0551400	1.418	406	2	22.4	Rossmann-like alpha/beta/alpha sandwich fold domain protein.
Os02g0767500	1.417	701	2	25.8	Mitochondrial phosphate transporter.
Os09g0542000	1.416	388	4	14.9	Similar to Ribonuclease P.
Os06g0247800	1.415	863	4	21.6	Similar to Dynamin-like protein (Fragment).
Os03g0802700	1.415	235	6	10.5	RH27 helicase (Fragment).
Os12g0506400	1.415	95	1	6.7	Cornichon family protein.
Os01g0276700	1.414	1642	14	38.8	Similar to Pyruvate kinase, cytosolic isozyme.

Os01g0883900	1.413	305	13	21.7	DUF248, methyltransferase putative family protein.
Os07g0622200	1.41	786	9	25.7	Similar to M-160-u1_1 (Fragment).
Os03g0718100	1.409	3081	2	41.9	Actin 1.
Os06g0133800	1.408	1492	12	28.6	Similar to Transferase.
Os07g0539100	1.408	619	8	22.6	Glycoside hydrolase, family 17 protein.
Os10g0564300	1.407	714	3	28.7	Similar to 60S ribosomal protein L27.
Os12g0605400	1.406	244	4	33.6	Similar to CROC-1-like protein (Fragment).
Os12g0443700	1.405	1191	16	39.2	Similar to Glu-prolyl-tRNA aminoacyl synthetase (Fragment).
Os03g0700400	1.399	1056	3	37.3	Similar to LOX4 (Fragment).
Os03g0688200	1.392	463	4	13.5	Conserved hypothetical protein.
Os11g0104900	1.392	1236	16	23.7	Similar to Clathrin heavy chain.
Os08g0117300	1.39	2212	3	57	Similar to 40S ribosomal protein S13.
Os04g0249600	1.389	151	2	23.9	Rhodanese-like domain containing protein.
Os07g0109500	1.388	504	4	31.6	Ribosomal protein L13 family protein.
Os02g0182500	1.386	670	1	29.4	Similar to Proteasome subunit beta type 3.
Os05g0399100	1.385	290	3	14.2	Similar to Endo-1,3;1,4-beta-D-glucanase.
Os03g0703100	1.384	265	6	13.7	Similar to Beta-glucosidase.
Os08g0555200	1.381	238	3	6.7	Nonaspanin (TM9SF) family protein.
Os03g0158500	1.38	172	5	7.6	YT521-B-like protein family protein.
Os11g0171500	1.38	593	7	27.9	Similar to Calcium-dependent protein kinase SK5 (CDPK).
Os07g0603100	1.378	149	2	5.1	Nucleotide-binding, alpha-beta plait domain containing protein.
Os03g0577000	1.377	1613	6	45.6	Similar to Ribosomal protein S3 (Fragment).
Os08g0162000	1.377	73	2	14.6	Similar to transmembrane 9 superfamily protein member 1.
Os01g0938200	1.376	142	4	9.6	Similar to RNA-binding protein BRUNOL5 (Fragment).
Os10g0377400	1.375	693	6	33.8	Similar to Ras-related protein Rab11D.
Os12g0207300	1.375	52	2	12.7	Similar to Clathrin coat assembly protein AP17.

Os03g0693900	1.375	111	3	13.2	Similar to Oxalate oxidase 1 (EC 1.2.3.4) (Germin).
Os03g0212800	1.375	262	5	13.4	Similar to Beta-glucosidase.
Os10g0573800	1.373	669	6	34.8	Mitochondrial carnitine/acylcarnitine carrier-like protein.
Os03g0276500	1.371	9562	9	47.5	Similar to Heat shock protein 70.
Os05g0539700	1.37	727	9	28	Similar to Nucleosome assembly protein 1.
Os07g0614500	1.37	1784	7	59.4	Similar to Elongation factor 1 beta 2.
Os02g0785800	1.37	356	2	37.5	Similar to Ribosomal protein L35A.
Os07g0636000	1.366	1977	4	32.5	Similar to H/ACA ribonucleoprotein complex subunit 4.
Os06g0671900	1.365	10005	2	58.5	Similar to Tubulin beta-3 chain.
Os03g0231600	1.365	241	3	9.4	Similar to Branched-chain-amino-acid aminotransferase 3.
Os06g0163200	1.365	64	2	5.9	Esterase/lipase/thioesterase domain containing protein.
Os07g0114400	1.364	246	6	25.8	Casein kinase II alpha subunit.
Os02g0313400	1.364	227	3	11.4	Apoptosis inhibitory 5 family protein.
Os02g0602500	1.364	75	4	16.5	Similar to Pre-mRNA-splicing factor ISY1.
Os09g0375000	1.364	439	6	18.6	Enolase (EC 4.2.1.11) (Fragment).
Os05g0558000	1.363	408	8	30.1	Ribosomal protein L1 family protein.
Os06g0574450	1.363	90	2	5.8	Hypothetical conserved gene.
Os05g0103100	1.36	788	3	23.1	Translocon-associated beta family protein.
Os03g0757900	1.359	4804	5	65	Similar to UDP-glucose 6-dehydrogenase.
Os02g0576700	1.359	857	8	44	Nucleosome assembly protein (NAP) family protein.
Os06g0308300	1.352	571	6	15.5	Similar to 65kD microtubule associated protein.
Os03g0333300	1.35	414	2	39.3	Similar to eIF-2-beta (P38).
Os12g0623900	1.35	9641	7	48.8	Similar to Ethylene-responsive methionine synthase (Fragment).
Os10g0530900	1.349	458	4	25.8	Similar to Glutathione S-transferase GST 30.
Os04g0497200	1.348	169	4	9.3	Cellulase precursor.
Os09g0497400	1.345	149	2	12.4	DUF850, transmembrane eukaryotic family protein.

Os01g0627500	1.343	66	4	7.3	Cytochrome P450 family protein.
Os08g0532900	1.343	156	1	6.1	emp24/gp25L/p24 family protein.
Os01g0708100	1.343	239	9	21.5	Similar to N-myristoyl transferase.
Os02g0821800	1.343	1627	8	42.9	Similar to Fibrillarin-2.
Os03g0836200	1.342	137	2	12.7	Similar to RNA-binding protein RZ-1.
Os05g0235800	1.339	331	6	10.8	DNA-dependent ATPase MCM domain containing protein.
Os07g0613200	1.339	380	3	28.1	Similar to 60S ribosomal protein L27a-3.
Os11g0240600	1.336	235	2	7.7	Alpha/beta hydrolase fold-3 domain containing protein.
Os05g0134400	1.336	1288	6	26.3	Similar to peroxidase 1.
Os04g0107600	1.335	1423	11	27.9	Arginine decarboxylase.
Os08g0113100	1.335	3729	16	60.4	Similar to Fructokinase (Fragment).
Os02g0102700	1.335	470	3	25.8	Similar to AGL157Cp.
Os09g0497700	1.333	253	5	15.5	Autophagy protein 16 domain containing protein.
Os03g0181500	1.333	551	6	15.3	Similar to Fiddlehead protein.
Os01g0874700	1.331	1087	12	34.3	GOLD domain containing protein.
Os07g0624700	1.331	383	3	28.6	UMP/CMP kinase a (EC 2.7.1.48).
Os05g0243300	1.33	149	2	11	Similar to 50S ribosomal protein L13.
Os04g0483500	1.327	389	6	23	Similar to B-keto acyl reductase.
Os04g0504800	1.325	431	6	13.5	Similar to Poly(A)-binding protein.
Os02g0541325	1.324	85	2	5.6	Similar to Serine decarboxylase.
Os03g0599800	1.323	4641	11	58.8	Reversibly glycosylated polypeptide.
Os08g0500700	1.323	4516	8	40.6	Similar to Heat shock protein 82.
Os03g0749300	1.319	851	8	27.2	Similar to Exoglucanase precursor.
Os01g0294700	1.317	707	8	33.7	Haem peroxidase, plant/fungal/bacterial family protein.
Os03g0268400	1.316	100	4	13.3	Similar to Mannose-1-phosphate guanyltransferase.
Os05g0574500	1.316	568	2	32.6	Similar to GTP-binding nuclear protein Ran1B (Fragment).

Os03g0648400	1.314	832	3	18	Similar to DnaJ protein homolog (DNAJ-1).
Os01g0814900	1.314	611	5	23.7	Similar to Cytochrome b5 reductase.
Os05g0482700	1.313	1878	9	38.8	Similar to 2,3-bisphosphoglycerate-independent phosphoglycerate.
Os04g0618500	1.313	98	3	14.1	Similar to Gamma-SNAP (Fragment).
Os06g0598900	1.31	479	7	30.7	Similar to Serine-threonine kinase receptor-associated protein.
Os07g0448800	1.309	531	5	18.3	Aquaporin.
Os07g0671700	1.309	130	4	9.8	Similar to Arginine methyltransferase-like protein.
Os10g0533600	1.309	119	3	12.5	Similar to Mitogen-activated protein kinase homolog MMK2.
Os03g0733400	1.307	449	4	20.8	Zinc finger, BED-type predicted domain containing protein.
Os09g0237600	1.306	349	5	18.7	Serine/threonine protein kinase-related domain protein.
Os05g0186300	1.306	1436	6	27.9	Similar to NADP-malic enzyme.
Os03g0780400	1.306	335	4	43.4	Similar to Actin-depolymerizing factor 6 (ADF-6) (AtADF6).
Os02g0739600	1.305	662	9	34.4	Similar to Pyruvate dehydrogenase E1 component alpha subunit.
Os02g0498700	1.304	61	2	6.6	Similar to QUA1.
Os05g0373700	1.304	1231	2	43.4	Similar to NAC-alpha-like protein 3.
Os05g0155100	1.303	790	5	33.5	Similar to 60S ribosomal protein L18.
Os03g0750700	1.302	399	9	37.8	Six-bladed beta-propeller, TolB-like domain containing protein.
Os01g0323600	1.298	2002	4	52	Similar to S-adenosylmethionine synthase 2.
Os02g0240100	1.298	568	5	19	Similar to Peroxidase 2 (Fragment).
Os02g0529600	1.298	141	5	16	Similar to Xyloglucan 6-xylosyltransferase (AtXT1).
Os07g0546000	1.298	672	4	42.4	Similar to Isopentenyl pyrophosphate:dimethyllallyl pyrophosphate isomerase (Fragment).
Os05g0400700	1.296	81	3	20.9	Similar to DNA-directed RNA polymerase II 19 kDa polypeptide.
Os09g0565200	1.294	75	2	7.8	Similar to Nucleic acid-binding protein precursor.
Os02g0580300	1.293	4913	3	63	Similar to 14-3-3 protein 6.
Os01g0617500	1.293	1118	19	24.3	Tetratricopeptide-like helical domain containing protein.

Os02g0247200	1.292	367	4	16.5	Similar to T-complex protein 1, gamma subunit (TCP-1-gamma).
Os04g0459700	1.292	274	6	36.7	Similar to H0219H12.3 protein.
Os11g0432900	1.291	305	7	21.3	Peptidase S10, serine carboxypeptidase family protein.
Os08g0465800	1.291	952	8	33.4	Similar to Glutamate decarboxylase.
Os06g0680700	1.291	220	7	15.1	Cytochrome P450 family protein.
Os03g0402000	1.29	327	6	35.3	TRAPP I complex, Bet3 domain containing protein.
Os03g0105600	1.29	8901	4	52.6	Tubulin beta-1 chain (Beta-1 tubulin).
Os02g0497700	1.288	285	4	11.7	Similar to Ras-GTPase-activating protein.
Os07g0616600	1.287	2524	4	48.5	Similar to 40S ribosomal protein SA (p40).
Os07g0440100	1.287	995	6	29.4	Conserved hypothetical protein.
Os01g0871300	1.287	407	8	24.7	Pyridoxal phosphate-dependent transferase.
Os05g0543400	1.286	364	5	27.3	Similar to Farnesyl diphosphate synthase (Fragment).
Os06g0157000	1.286	534	5	21.4	Esterase, SGNH hydrolase-type domain containing protein.
Os05g0533100	1.282	393	3	18.5	Similar to plasminogen activator inhibitor 1 RNA-binding protein.
Os11g0585700	1.282	130	3	14.7	Similar to 50S ribosomal protein L4.
Os12g0568800	1.28	505	7	17.9	Similar to predicted protein.
Os10g0516400	1.28	604	8	38.8	Conserved hypothetical protein.
Os03g0236200	1.278	341	2	20.1	Similar to Glutamate decarboxylase isozyme 3.
Os12g0489100	1.278	101	3	6.7	Similar to Ubiquitin-specific protease 12.
Os01g0570700	1.276	360	4	14.4	Carbamoyl-phosphate synthetase, large subunit.
Os02g0177600	1.276	289	5	17.5	Similar to 4-coumarate--CoA ligase 1 (Fragment).
Os08g0459300	1.276	375	2	19	Conserved hypothetical protein.
Os04g0473400	1.275	1310	7	42.8	Similar to 60S ribosomal protein L6-B (L17) (YL16) (RP18).
Os06g0167000	1.274	172	9	9.9	Similar to PRP8 protein (Fragment).
Os02g0554900	1.273	110	4	9	Similar to Protein disulfide isomerase (Fragment).
Os02g0626100	1.273	4962	18	47.1	Similar to Phenylalanine ammonia-lyase.

Os08g0162100	1.272	225	5	11.4	Similar to CTV.2.
Os05g0363200	1.272	1452	7	26.8	UDP-glucuronic acid decarboxylase.
Os03g0711400	1.27	3582	6	37.2	Similar to Coatomer alpha subunit.
Os05g0531200	1.27	362	4	27.7	Pollen Ole e 1 allergen and extensin domain containing protein.
Os11g0455800	1.27	1522	12	40.1	Similar to Serine hydroxymethyltransferase.
Os03g0807700	1.269	1648	10	35.9	Protein of unknown function DUF642 domain containing protein.
Os03g0791800	1.269	103	1	11.2	Rad6 (Ubiquitin carrier protein).
Os07g0600400	1.268	126	5	13.8	WD40/YVTN repeat-like domain containing protein.
Os06g0701100	1.268	3807	4	52.7	Eukaryotic initiation factor 4A (eIF4A) (eIF-4A).
Os07g0688800	1.266	248	4	8.5	Aldehyde dehydrogenase domain containing protein.
Os01g0654500	1.266	1996	14	47.8	Similar to NADP-isocitrate dehydrogenase.
Os01g0899425	1.266	1115	1	67	Similar to ribulose 1,5-bisphosphate carboxylase large subunit.
Os03g0341100	1.265	506	4	28.3	Similar to 60S ribosomal protein L18.
Os04g0103200	1.265	1120	16	46.4	Proteasome component region PCI domain containing protein.
Os03g0406200	1.265	864	7	67.7	Elongation factor 1 beta 2.
Os06g0127000	1.264	335	2	10.7	Peroxisomal biogenesis factor 11 family protein.
Os12g0145100	1.264	244	4	40.8	Similar to Wali7 protein (Fragment).
Os05g0108800	1.261	652	3	52.6	Cytochrome b5.
Os03g0424500	1.258	1103	7	42.5	Similar to 40S ribosomal protein S19-3.
Os04g0486600	1.258	10092	8	65	Similar to Glyceraldehyde-3-phosphate dehydrogenase, cytosolic 3.
Os04g0669800	1.256	441	10	32.6	Methylthioribose kinase.
Os09g0485900	1.256	1741	1	41.1	Similar to 60S ribosomal protein L9 (Gibberellin-regulated protein).
Os07g0683900	1.253	799	7	32.1	Ricin B-related lectin domain containing protein.
Os12g0624000	1.252	10001	7	48.8	Similar to Methionine synthase protein.
Os08g0430500	1.251	4028	6	62.9	Similar to 14-3-3-like protein S94.
Os10g0569200	1.25	1125	21	35.2	Six-bladed beta-propeller, TolB-like domain containing protein.

Os04g0485000	1.249	781	5	19	Similar to 26S proteasome subunit RPN7.
Os01g0532200	1.247	240	8	20.6	FF domain domain containing protein.
Os03g0209000	1.246	123	2	12.3	Similar to MFP1 attachment factor 1.
Os03g0335300	1.245	193	4	7.3	Similar to Vacuolar sorting receptor homolog (Fragment).
Os01g0342900	1.245	730	12	28.8	Similar to Adenosine monophosphate binding protein 1 AMPBP1.
Os07g0689300	1.244	208	5	17.2	Similar to Lon protease homolog, mitochondrial.
Os09g0539800	1.244	739	3	54.2	Similar to Acyl carrier protein III, chloroplast precursor (ACP III).
Os09g0415700	1.242	456	5	19.5	DUF248, methyltransferase putative family protein.
Os08g0240800	1.241	222	7	9.5	Similar to Actin filament bundling protein P-115-ABP.
Os04g0446300	1.241	211	1	6.2	Microsomal signal peptidase 25 kDa subunit family protein.
Os04g0372800	1.239	596	5	16.7	Nucleotide-binding, alpha-beta plait domain containing protein.
Os08g0321000	1.238	649	5	16.6	FAS1 domain domain containing protein.
Os03g0628900	1.238	332	3	26.2	Prefoldin domain containing protein.
Os06g0571400	1.236	1585	4	30.8	Similar to Elongation factor 1 gamma-like protein (Fragment).
Os03g0713000	1.235	589	11	28.4	Similar to Chloroplast threonine deaminase 1.
Os07g0204500	1.234	87	2	6.6	Survival protein SurE family protein.
Os03g0767000	1.233	266	10	22.1	Similar to Allene oxide synthase.
Os03g0182700	1.233	666	6	30.5	Eukaryotic translation initiation factor 3 subunit 12 (eIF3k).
Os08g0112800	1.232	207	4	40.1	Rickettsia 17 kDa surface antigen family protein.
Os04g0556300	1.232	139	2	17.3	Glutathione peroxidase.
Os10g0551800	1.231	397	1	10.3	Root-specific protein (RCc2 protein).
Os02g0287000	1.231	1605	4	48.3	Similar to 40S ribosomal protein S3a (CYC07 protein).
Os02g0503400	1.229	99	2	13.8	Similar to 60S ribosomal protein L35.
Os11g0256050	1.225	177	4	22.1	Hypothetical conserved gene.
Os01g0191500	1.225	581	11	36	Similar to Mitochondrial processing peptidase.
Os03g0264400	1.225	186	4	11.2	Anthranilate synthase alpha 2 subunit.

Os02g0672200	1.225	493	7	17.9	Similar to AGO1 homologous protein.
Os01g0710000	1.224	622	9	35.1	Similar to WD-repeat protein RBAP1.
Os05g0512600	1.224	365	2	15.4	X8 domain containing protein.
Os03g0112101	1.224	176	6	15.4	Similar to Adaptin N terminal region family protein, expressed.
Os06g0695300	1.224	460	5	27.1	Haem peroxidase, plant/fungal/bacterial family protein.
Os11g0525200	1.224	1142	15	34.3	Similar to Cytochrome P450 51.
Os03g0177400	1.223	7049	13	46.5	EF-1 alpha.
Os12g0566300	1.223	883	6	25.1	Similar to ATP citrate lyase beta (Fragment).
Os03g0717600	1.222	288	7	17.2	Zinc finger, C2H2-type matrin domain containing protein.
Os03g0198400	1.222	682	11	27.9	Similar to Seryl-tRNA synthetase (SerRS) (Fragment).
Os09g0407950	1.222	257	7	10.7	Similar to transducin family protein / WD-40 repeat family protein.
Os02g0173500	1.222	213	4	17.1	Similar to Cholinephosphate cytidylyltransferase.
Os06g0300800	1.222	312	6	9.3	Hypothetical conserved gene.
Os10g0355800	1.221	2143	4	38.1	Similar to ATP synthase CF1 beta subunit.
Os03g0283100	1.221	554	7	38.1	Similar to In2-1 protein.
Os05g0320700	1.22	701	17	42	Similar to Cytochrome P450.
Os01g0300200	1.217	2223	20	41.4	Similar to ATP-citrate lyase subunit B.
Os04g0620700	1.216	1213	8	15.8	Nucleotide-binding, alpha-beta plait domain containing protein.
Os01g0235400	1.215	451	6	17.3	Similar to Importin-alpha re-exporter.
Os01g0290100	1.215	196	5	14.7	Pyridoxal phosphate-dependent transferase.
Os04g0397100	1.214	678	3	40.2	Similar to ClpC protease.
Os05g0585900	1.214	240	5	20.3	Mitochondrial carrier protein domain containing protein.
Os05g0432400	1.214	201	4	9.7	Hypothetical conserved gene.
Os03g0807600	1.212	489	3	19.5	Similar to Cyclopropane-fatty-acyl-phospholipid synthase.
Os03g0221500	1.211	398	3	7.5	Glycoside hydrolase, family 17 protein.
Os09g0378300	1.211	826	7	20.1	Similar to Leucyl-tRNA synthetase, cytoplasmic (LeuRS).

Os05g0181000	1.209	1413	11	27.3	Heat shock protein Hsp70 family protein.
Os03g0141000	1.207	119	4	33.8	60S ribosomal protein L21.
Os05g0168400	1.206	121	5	26	Conserved hypothetical protein.
Os07g0694700	1.205	1771	7	52.2	L-ascorbate peroxidase.
Os09g0515200	1.205	1437	10	48.8	Beta 7 subunit of 20S proteasome.
Os01g0895600	1.204	467	6	28.8	Similar to Calreticulin-3.
Os03g0854300	1.201	83	3	7.5	Nucleotide-binding, alpha-beta plait domain containing protein.

Supplementary Table 5. List of common target proteins in embryos of germinated rice seeds in response to both BR-deficiency and BR-insensitivity by iTRAQ (fold-change criterion ≥1.5 or ≤0.67)

Gene ID	NIP(BRZ / Mock) fold change	d61/NIP fold change	Protein score	Unique Peptide	Sequence coverage(%)	Description
Os02g0249000	0.406	0.411	804	10	22.7	Glutelin, Seed strage protein
Os02g0249600	0.568	0.57	3780	1	34.9	Similar to Glutelin.
Os02g0249800	0.452	0.45	3900	2	36.3	Glutelin precursor. >Os02t0249900-01 Glutelin precursor.
Os02g0453600	0.459	0.414	539	5	28.1	Similar to Glutelin.
Os02g0765600	0.576	0.561	660	9	32.5	Alpha-amylase glycoprotein, Degradation of starch granule
Os03g0734200	0.639	0.566	254	1	17.4	Conserved hypothetical protein.
Os05g0268500	0.451	0.369	131	5	12	Similar to Serine carboxypeptidase 2.
Os05g0329100	0.555	0.421	917	3	42.7	Prolamin.
Os06g0133000	0.482	0.57	495	9	22.2	Granule-bound starch synthase I, chloroplast precursor
Os07g0214300	0.474	0.321	1815	2	28.3	Seed allergenic protein RAG2 precursor.
Os07g0214600	0.465	0.383	606	3	25.5	Similar to Seed allergenic protein RA17 precursor.
Os07g0237100	0.667	0.59	121	2	8.5	RNA recognition motif domain domain containing protein.

Os01g0815800	2.061	2.224	682	1	19.8	Similar to 60S ribosomal protein L24-A (L30A) (RP29).
Os03g0799000	1.997	2.018	126	2	18.4	Similar to Histone H1.
Os02g0675700	1.741	1.617	236	4	18.4	methyltransferase putative family protein.
Os01g0880800	1.585	1.835	891	5	16.8	Similar to Acyl-[acyl-carrier-protein] desaturase
Os06g0705400	1.607	0.609	205	1	11.7	Similar to Nonspecific lipid-transfer protein 2P.
Os09g0127700	1.506	0.4	221	2	21.5	Conserved hypothetical protein.

Supplementary Table 6. List of primers used for quantitative real-time PCR assay

Primer name	Primer sequence (5’-3’)
RAmy1AqRT-F	CATACATCCTCACCCACCCCG
RAmy1AqRT-R	ATCCCCTGCCGGTTTCTGAT
MFT2qRT-F	CCGCAACGACCTCTACACCC
MFT2qRT-F	CTCGTACAGCACCAGCACGTA
HDqRT-F	TCCCTCACCTGAAGAAGACGC
HDqRT-R	GGTGATCTCCGACTCGACCA
PKqRT-F	GCTGTCTGGTGAAACTGCCC
PKqRT-R	CCTCGTTGAGCAGAGCCTGA
SBE1qRT-F	CAAAACACAAGATGGAGAAGGATA
SBE1qRT-R	TTGTGAGGGATGGCAGGCT
LOX3qRT-F	CTGTCGCTGGTGGAGCAGAT
LOX3qRT-R	CGCCTTGATCGAGTAGCCCA
UBCqRT-F	CCGTTTGTAGAGCCATAATTGCA
UBCqRT-R	AGGTTGCCTGAGTCACAGTTAAGTG
